# RiboBA: a bias-aware probabilistic framework for robust ORF identification across diverse ribosome profiling protocols

**DOI:** 10.1093/nar/gkag969

**Published:** 2026-10-14

**Authors:** Junyu Bai, Ruolin Yang

**Affiliations:** College of Life Sciences, Northwest A&F University, Yangling, Shaanxi 712100, China; College of Life Sciences, Northwest A&F University, Yangling, Shaanxi 712100, China

## Abstract

By mapping ribosome-protected fragments genome-wide, ribosome profiling (Ribo-seq) has uncovered extensive translation beyond conventional coding sequences, revealing non-canonical ORFs (ncORFs) with roles in diverse biological processes. However, robust ncORF identification remains challenging when Ribo-seq datasets exhibit attenuated periodicity. Protocol-induced biases during library construction is an important contributor to this loss, yet most ORF callers do not explicitly model these distortions. Here, we present RiboBA, a bias-aware probabilistic framework for robust ORF identification from Ribo-seq data. RiboBA combines a generative module that jointly infers protocol-induced biases and codon-level ribosome occupancy with a supervised module that identifies translated ORFs and initiation sites from the bias-adjusted profiles. In simulations with known P-site positions, RiboBA improved exact nucleotide- and codon-level P-site assignment relative to a conventional fixed-offset approach. Across simulations and diverse empirical Ribo-seq datasets, together with orthogonal immunopeptidomics evidence, RiboBA accurately recovers protocol-induced parameters and improves ncORF identification, with pronounced advantages for RNase I libraries with attenuated periodicity, and for MNase and nuclease P1 datasets, while maintaining competitive runtimes. In a *Drosophila* case study, RiboBA identified candidate ncORFs with coding potential, including recurrent upstream translation in *ThrRS* and *Mettl2*, linking ncORF translation to threonine-related translational regulation.

## Graphical Abstract

**Graphical Abstract:**
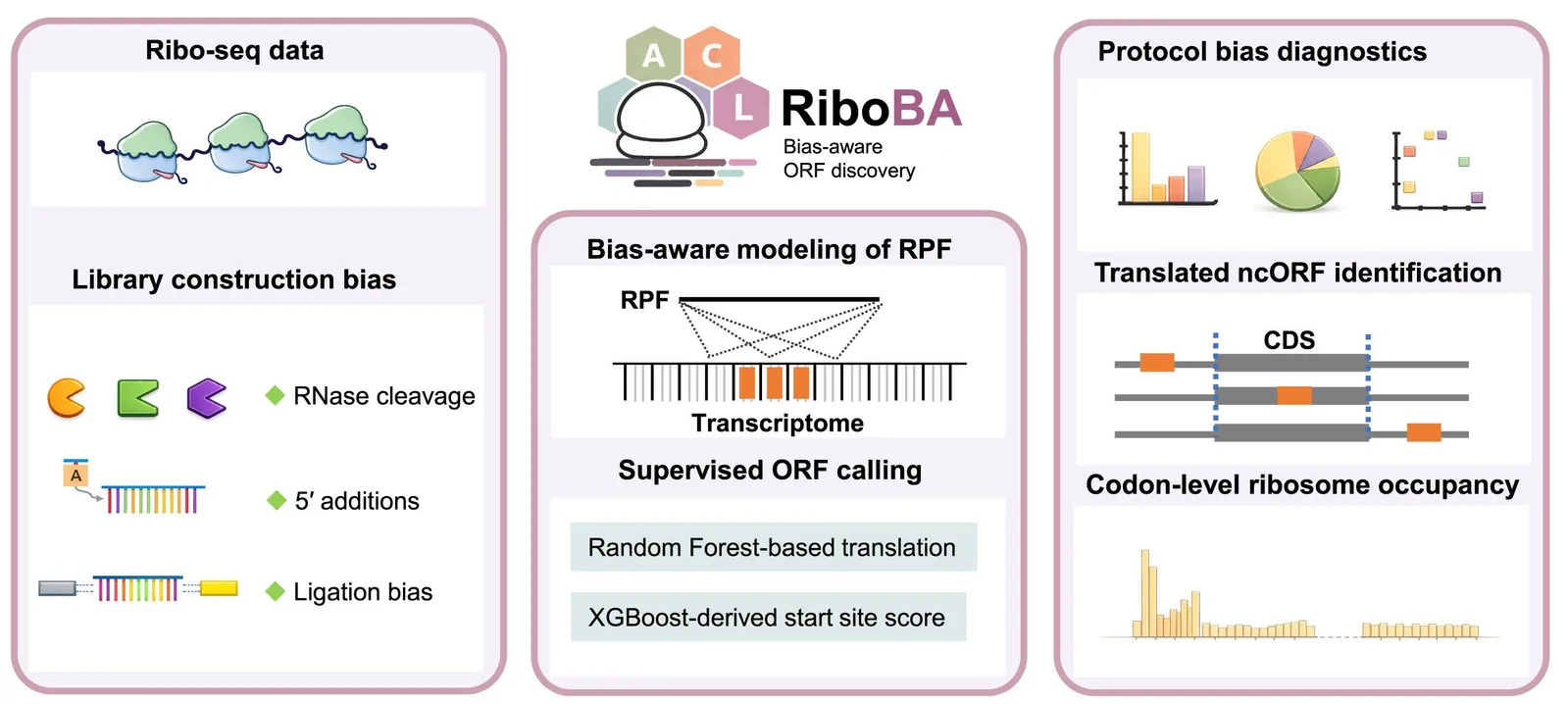

## Introduction

Ribosome profiling (Ribo-seq) captures genome-wide snapshots of translation by nuclease digestion of unprotected mRNA followed by deep sequencing of ribosome-protected fragments (RPFs) [1, 2]. Computational analysis of RPF signals enables transcriptome-wide identification of actively translated open reading frames (ORFs) and has revealed extensive translation beyond canonical gene annotations, giving rise to non-canonical ORFs (ncORFs) [3–5]. These ncORFs arise in diverse genomic contexts [6]. Large-scale studies combining Ribo-seq with CRISPR-based screens have revealed that dozens to hundreds of ncORFs are essential for cell proliferation [5] and stress responses [7], and in some cases act through encoded microproteins involved in signaling, metabolic rewiring, and disease-related pathways [8]. In parallel, immunopeptidomic analyses have revealed that a substantial fraction of MHC class I-presented peptides originate from ncORFs, supporting their potential to generate antigenic peptides and contribute to immune recognition [9]. Despite their biological relevance [10], many ncORFs are short and often translated at relatively low levels [11], making them inherently difficult to identify with confidence [12].

Since the advent of Ribo-seq, considerable effort has therefore been devoted to developing computational methods for ncORF identification [13, 14]. These methods typically leverage characteristic signatures of translation inferred by mapping RPFs to the peptidyl (P)-site positions [13]. Computational approaches for identifying translated ncORFs can be broadly categorized into three methodological classes: (i) supervised classifiers, such as ORF-RATER [15] and RibORF [16], which apply regression or tree-based models to features reflecting periodicity and read coverage; (ii) generative probabilistic models, including PRICE [17] and Rp-Bp [18], which model codon-level translation activity using expectation–maximization or Bayesian inference; and (iii) periodicity-based statistical methods, such as RiboTaper [19], RiboCode [20], RiboTISH [21], and SPECtre [22], which directly assess 3-nt periodicity via spectral analysis or frame-specific statistical testing.

Although these methods differ in their downstream statistical frameworks, they generally share a common preprocessing strategy: P-site positions are assigned using fixed, length-specific offsets. This strategy introduces two major limitations. First, it typically restricts analysis to a subset of RPF lengths with strong periodicity and applies offset estimates that differ across tools [23], which may lead to signal loss and distortion. Second, simplifying RPFs to a fixed-offset representation at the outset discards protocol-dependent information that could otherwise be modeled downstream. PRICE alleviates the first limitation by using probabilistic P-site assignment and retaining all RPF lengths, but it does not explicitly account for protocol-induced biases. A growing body of evidence demonstrates that systematic biases introduced during Ribo-seq library construction can profoundly distort RPF signals [24–27]. Nuclease digestion bias alters RPF length and position: under-digestion produces extended overhangs that broaden the distribution of RPF lengths and obscure 3-nt periodicity [28], whereas over-digestion—particularly with RNase I—can dissociate ribosomal subunits and alter the distribution of RPFs [29]. Alternative nucleases such as MNase and P1 can reduce over-digestion [30, 31]. However, MNase introduces cleavage bias [32], and the impact of these biases remains poorly addressed by existing ORF-calling methods. Ligation and circularization steps introduce additional biases, as enzymes such as T4 RNA ligase favor specific terminal nucleotides, resulting in a skewed representation of RPFs [33–35]. Furthermore, 5$^\prime$ non-templated nucleotide additions introduced by reverse transcriptase cause systematic offset errors that disrupt 3-nt periodicity [36]. These biases may partly underlie the substantial discrepancies and limited reproducibility in ncORF identification reported across ORF-calling tools in recent benchmarking studies [37, 38].

To address these challenges, we developed RiboBA, a bias-aware probabilistic framework that explicitly models systematic biases in Ribo-seq data to improve ORF identification. RiboBA models footprint generation as a probabilistic mapping from latent P-site positions to observed RPFs, incorporating protocol-induced biases—including nuclease cleavage bias, the ribosome protection effect, ligation bias, and 5$^\prime$ non-templated nucleotide additions—within a unified framework. The parameters associated with these protocol-induced biases are jointly inferred with codon-level ribosome occupancy using an expectation–maximization-like optimization strategy. RiboBA performs probabilistic P-site mapping by distributing each RPF across all geometrically compatible positions, weighting them by their posterior probabilities rather than assigning a single fixed site. By reweighting frame-ambiguous signals, this soft assignment corrects protocol-induced distortions and restores attenuated 3-nt periodicity. Leveraging adjusted P-site profiles, RiboBA computes translation-relevant features for both ORFs and initiation sites, which are evaluated via supervised learning models.

Using bias-aware simulations and analyses of human and *Drosophila* Ribo-seq datasets across multiple protocols, we show that RiboBA improves both canonical and ncORF detection compared to five state-of-the-art tools. (i) In simulations informed by empirical parameters derived from RNase I, MNase, and nuclease P1, RiboBA consistently achieved superior ORF detection performance. (ii) Across nine human datasets, RiboBA yielded highly reproducible ncORF detections with superior replicate concordance and support from orthogonal immunopeptidomics evidence across multiple biotypes. (iii) RiboBA robustly identifies conserved ncORFs with coding potential in *Drosophila melanogaster* MNase datasets, confirming its adaptability across distinct protocols. Despite its sophisticated modeling, RiboBA remains computationally efficient on large-scale datasets while providing bias metrics and ribosome occupancy profiles for downstream analysis.

## Materials and methods

### Reference construction and ORF search space

RiboBA constructs a transcript-level reference from the input genome and gene annotation. For each gene, RiboBA selects a single representative transcript using a deterministic rule that prioritizes the transcript with the longest coding sequence and resolves remaining ties by total transcript length. This strategy provides a consistent reference for ORF analysis while reducing ambiguity in read assignment caused by extensive exon sharing among transcript isoforms. Candidate ORFs are then exhaustively enumerated on each representative transcript by scanning for all candidate start codons and extending to the first in-frame stop codon (UAA, UAG, or UGA).

### Probabilistic modeling of RPF generation

RiboBA models RPF generation as a protocol-dependent transformation from latent ribosome P-site positions to observed reads. Aligned reads are collapsed into footprint classes $r \equiv (s, \ell )$, defined by their 5$^\prime$ terminal coordinate *s* and fragment length *ℓ*, with observed counts denoted as $y_r$. To estimate protocol-induced bias parameters *Θ*, the model is fit using well-characterized, annotated coding sequences. Let $\lambda _p$ represent the ribosome occupancy at candidate P-site *p*. The expected read count for class *r* is defined as a mixture over feasible P-sites:


(1) \begin{eqnarray*}
\widehat{y}_{r} \,\,=\,\, \sum _{p\in \mathcal {P}(r)} \lambda _p \, \Pr (r\mid p;\Theta ),
\end{eqnarray*}

where $\mathcal {P}(r)$ is the set of P-sites compatible with class *r* given ribosome-geometry constraints on the 5$^\prime$/3$^\prime$ end-to-P-site distances for length *ℓ* (Supplementary Note S1).

The conditional probability $\Pr (r\mid p;\Theta )$ is factorized as


(2) \begin{eqnarray*}
\Pr (r\mid p;\Theta ) \,\,=\,\, \frac{1}{Z(p)} \, A(r;\alpha ) \, C(r\mid p;\theta ) \, L(r;\beta ),
\end{eqnarray*}

where *Z(p)* is the normalization constant over all footprint classes $\mathcal {R}(p)$ compatible with P-site *p*. The factors *A*, *C*, and *L* represent 5$^\prime$ additions, nuclease cleavage, and ligation efficiency, respectively. Of these, only $C(r\mid p;\theta )$ depends on the candidate P-site *p* and is thus the factor that discriminates among candidate P-sites for a given read (together with the genome-wide P-site abundance). The 5$^\prime$-addition term *A(r;α )* still affects P-site inference indirectly, because estimating *α* recovers each read’s true 5$^\prime$ terminus and hence its length class, on which the candidate-P-site geometry and the distances $d_5, d_3$ are defined; the ligation term instead governs sequence-dependent fragment recovery rather than the P-site position of a given read.

Ribosome steric constraints define the admissible distances from P-site *p* to the 5$^\prime$ and 3$^\prime$ termini, denoted $d_5$ and $d_3$. Assuming conditional independence between termini given *p*:


(3) \begin{eqnarray*}
C(r\mid p;\theta ) \,\,=\,\, P_5(d_5 \mid p) \times P_3(d_3 \mid p).
\end{eqnarray*}

These probabilities capture both sequence preferences and distance-dependent protection effects. Let $x_t(p,d)$ be the nucleotide at distance *d* on terminus $t \in \lbrace 5,3\rbrace$, $b_x$ the nucleotide-specific bias, and $h_t(d)$ the distance-dependent protection. The maintenance probability at distance *d* is defined as $m_t(d \mid p) = b_{x_t(p,d)}^{h_t(d)}$. To model cleavage at distance *d*, we use a discrete hazard formulation over the admissible set $\mathcal {D}_t$:


(4) \begin{eqnarray*}
P_t(d \mid p) \propto \bigl (1 - m_t(d \mid p)\bigr ) \prod _{k \in \mathcal {D}_t, \, k < d} m_t(k \mid p), \quad d \in \mathcal {D}_t.
\end{eqnarray*}

The normalization ensures $\sum _{d \in \mathcal {D}_t} P_t(d \mid p) = 1$.

Protocol-dependent 5$^\prime$ additions, observed as first-base mismatches, are modeled by a library-specific categorical distribution *α* over $\mathcal {A} = \lbrace A, C, G, T, \varnothing \rbrace$, where *∅* denotes no addition.

To account for sequence-dependent fragment recovery, ligation efficiency is modeled as the product of terminus-specific probabilities:


(5) \begin{eqnarray*}
L(r;\beta ) = \beta _5(u) \times \beta _3(v),
\end{eqnarray*}

where $\beta _5(u)$ and $\beta _3(v)$ are ligation probabilities associated with 5$^\prime$ and 3$^\prime$ terminal *k*-mers *u* and *v* extracted from class *r*.

### Inference of model parameters and ribosome occupancies

Model parameters and P-site occupancies are inferred via a sequential procedure. First, the distribution of 5$^\prime$ additions *α* is estimated by modeling mismatch compositions at the first aligned position relative to the reference. This approach recovers the full addition distribution, including cases where added bases coincide with the reference (Supplementary Note S2). The estimated *α* is then used to construct reweighted footprint counts $\widetilde{y}_{r}$.

We then infer P-site occupancies $\lambda _p$ and cleavage-related parameters *θ* using an EM-like alternating optimization procedure. Let $\mathcal {R}$ denote the set of retained footprint classes after filtering. For each $r\in \mathcal {R}$, the true P-site position $p\in \mathcal {P}(r)$ is treated as a latent variable. During this stage, the effects of ligation are held fixed, and we use $\Pr _0(r\mid p;\theta )$, the normalized conditional probability induced by the cleavage component [Equation (3)].

In the E-step, we compute the posterior probabilities over feasible P-sites:


(6) \begin{eqnarray*}
w_{r,p} = \frac{\lambda _{p}\, \Pr _0(r \mid p; \theta )}{\sum _{p^{\prime } \in \mathcal {P}(r)} \lambda _{p^{\prime }}\, \Pr _0(r \mid p^{\prime }; \theta )}, \qquad p \in \mathcal {P}(r).
\end{eqnarray*}

In the M-step, we update P-site occupancies by aggregating posterior-weighted reweighted counts:


(7) \begin{eqnarray*}
\lambda _{p}^{\mathrm{new}} = \sum _{r \in \mathcal {R}} \widetilde{y}_r\, w_{r,p}.
\end{eqnarray*}

We update cleavage parameters *θ*, including sequence preferences at cleavage sites and distance-dependent steric protection effects, by maximizing the expected complete-data objective under $w_{r,p}$ (Supplementary Note S3). The E- and M-steps are iterated until the parameter estimates converge.

After stabilizing $(\lambda , \theta )$, we estimate the ligation parameters *β* using the multiplicative terminal *k*-mer model [Equation (5)]. We first compute the model-predicted baseline expectation $\widehat{y}^{(0)}_{r}$ under the current estimates of $(\lambda , \theta )$ with ligation effects held fixed. We then estimate *β* using a regression model that treats the observed footprint-class counts as multiplicative deviations from $\widehat{y}^{(0)}_{r}$. We use a log-link and include $\log \widehat{y}^{(0)}_{r}$ as an offset to isolate systematic deviations attributable to terminal ligation biases (Supplementary Note S4).

### Supervised identification of translated ORFs

Using bias-aware occupancies *λ* estimated across all three reading frames, we performed supervised learning to identify translated regions and select the most probable initiation site within each region.

Annotated coding sequence (CDS) regions served as the primary positive training sets. To exclude weakly periodic or non-translating segments, we filtered these regions using a random forest (RF) classifier (ranger version 0.17.0, R) trained on well-supported CDS segments against an empirical null of randomly shifted read alignments. CDS segments passing this criterion formed the long-ORF positive set, while the long-ORF negative set combined these shift-based nulls with out-of-frame (*+1* and *-1*) CDS controls. To address the scarcity of validated non-canonical translation events, such as short ORFs (sORFs), we generated pseudo-sORFs from truncated long-ORF segments and pseudo-overlap sORFs by combining signals from overlapping structures.

To characterize these candidate regions, we extracted three feature classes: (i) coverage and strength, comprising the count and fraction of occupied codons and frame-stratified occupancy; (ii) 3-nt periodicity and frame consistency, using frame usage proportions, log-odds for dominant-frame preference, and frame entropy; and (iii) positional trends, including 5$^\prime$–3$^\prime$ signal gradients and frame-consistency scores relative to empirical CDS distributions.

The ranger random forest classifier was then trained on these features to assign translation probability scores, referred to here as RF translation scores. To account for heterogeneous background noise across ORF types, we applied class-specific decision thresholds. Candidates were categorized as CDS, uORF (upstream ORF), uoORF (upstream overlapping ORF), intORF (internal ORF), Ext (N-terminally extended CDS), Trunc (N-terminally truncated CDS), doORF (downstream overlapping ORF), dORF (downstream ORF), or Variant (novel transcript structures). Regions exceeding their respective thresholds were retained for initiation-site refinement.

To identify the most likely start codon within each translated region, we trained a binary XGBoost classifier (xgboost version 1.7.11.1, R). The training set utilized annotated CDS start sites as positives, with negatives derived from alternative in-frame ATGs located upstream or within the same CDS. Each candidate site was represented by (i) a 30-nt local P-site occupancy window and (ii) one-hot encoded flanking nucleotides. The decision threshold was optimized to maximize the F1 score on a held-out validation set. During inference, the first ATG exceeding this threshold was prioritized as the predicted initiation site; scoring of near-cognate start codons is also supported via user configuration.

Following ORF identification, we adjusted ribosome occupancy by restricting footprint assignment to in-frame positions within the detected ORFs. Finally, ORF coordinates were converted from transcript to genomic using annotated gene models. Each ORF was labeled by its position relative to annotated CDSs and flagged for in-frame overlap to facilitate downstream interpretation and visualization.

### Data processing and benchmarking

Human (HEK293/HEK293T) and *Drosophila* (embryo and S2 cell) Ribo-seq datasets were obtained from the NCBI SRA (Supplementary Table S1). Sequencing adapters were trimmed using Cutadapt (version 4.4), retaining 15–45 nt reads with base quality *≥*20 to capture the full spectrum of RPFs generated under different experimental protocols. Ribosomal RNA (rRNA) and transfer RNA (tRNA) reads were filtered by alignment to curated references using Bowtie (version 1.3.1) with up to two mismatches to remove contaminating reads with high sensitivity. Remaining reads were aligned to the RiboBA-defined representative transcriptome (see the “Reference construction and ORF search space” section) using a two-pass strategy: reads were first mapped to annotated protein-coding transcripts, followed by alignment of unmapped reads to annotated long non-coding transcripts (Bowtie, up to one mismatch). This staged approach reduces spurious alignments to long non-coding transcripts.

To benchmark RiboBA, we selected representative tools from three methodological classes: supervised classifiers (ORF-RATER [15], RibORF [16]), generative models (PRICE [17]), and periodicity-based methods (RiboCode [20], RiboTISH [21]). Analyses used the hg38 (Ensembl version 109) and BDGP6.32 (Ensembl version 6.32.54) references for human and *Drosophila*, respectively. Candidate ORFs included both canonical (ATG) and near-cognate initiators. Tool-specific configurations and full commands are detailed in Supplementary Note S5.

To enable cross-tool comparison, all predicted ORFs were converted to genomic coordinates and classified by their positions relative to annotated CDSs, with ORFs located on long non-coding transcripts designated as lncRNA-derived ORFs (lncORFs). Overlaps with annotated CDSs were assessed to determine frame-specific relationships; in-frame overlaps were excluded from downstream mass spectrometry validation to ensure the specificity of novel peptide identification.

### Simulation of ribosome profiling data

We simulated Ribo-seq libraries under three scenarios to evaluate parameter recovery and ORF detection.

To evaluate parameter recovery, we simulated Ribo-seq libraries using the probabilistic mixture model defined in Equation (1) under six protocol configurations: (i) RNase I (high dosage/high resolution), (ii) RNase I (low dosage/low resolution), (iii) MNase, (iv) nuclease P1, (v) RNase I with 5$^\prime$ additions, and (vi) RNase I with sequence-dependent ligation bias. Codon-level occupancies $\lambda _p$ were estimated from a representative HEK293T dataset (SRR23242344) and used as sampling probabilities in a multinomial distribution to simulate the placement of ribosomes across P-site positions. For each configuration, we simulated 10 replicate libraries of $1\times 10^6$ reads each, using 1000 randomly selected protein-coding genes.

To benchmark translation detection, we generated matched positive and negative sets through positional jittering of observed RPFs. Using RNase I (SRR23242345), MNase (SRR7073124), and nuclease P1 (SRR23242346) datasets, we selected 2000 protein-coding genes with robust CDS-aligned read support. The positive set retained original RPF coordinates, while the negative set was constructed by jittering RPF positions within *± 3* nt to disrupt 3-nt periodicity. The positive and negative RPF sets were combined with non-jittered reads from all other genes to form complete synthetic libraries, exported in FASTQ format with a constant Phred score of 40.

Finally, to assess ncORF detection, we simulated ncORF profiles by generating codon-level occupancies $\lambda _p$ from CDS-derived donor distributions. These profiles were mapped to candidate ncORF coordinates and then used to synthesize read libraries under five protocol regimes: RNase I, MNase, nuclease P1, RNase I with 5$^\prime$ additions, and RNase I with ligation bias. For each ncORF biotype, we generated a balanced set of 50 positive sets using bias parameters inferred from empirical datasets (SRR23242345, SRR7073124, SRR23242346). Negative sets were constructed using the same sampling procedure followed by nucleotide-scale P-site jittering to disrupt periodicity. These ncORF RPFs were combined with simulated CDS-derived background reads to form complete synthetic libraries, which were then exported in FASTQ format. RiboBA and alternative tools were evaluated using ROC and precision–recall (PR) curves via the precrec R package (version 0.14.0).

### Evolutionary conservation and coding potential analysis

Base-wise phyloP conservation scores [39] for *Drosophila melanogaster* (dm6, 27-way) were obtained from the UCSC Genome Browser. For each RiboBA-identified ORF, phyloP scores were extracted using rtracklayer (version 1.58.0), and the mean score was calculated as an ORF-level measure of evolutionary conservation.

To assess coding potential, PhyloCSF scores were extracted for ORFs *≥*10 codons from the Broad Institute dm6 PhyloCSF track hub, which provides strand- and frame-specific scores for the dm6 assembly [40]. For each candidate, we retained the score corresponding to its identified strand and reading frame as a metric of protein-coding signature.

### Immunopeptidomics validation of identified ORFs

Evidence for the translation of identified ORFs was evaluated using public HLA-I immunopeptidomics data from HEK293 cells (PXD025449). To ensure specificity, in-frame ncORFs overlapping annotated CDSs were excluded. For each tool, identified ORFs were collapsed into a non-redundant set by grouping those sharing a stop codon and retaining only the most upstream start site.

These sequences were translated *in silico* to construct custom protein databases for each tool and Ribo-seq sample. HLA-I spectra were queried against these databases using FragPipe (version 22.0) and MSFragger (version 4.1) under a non-specific HLA workflow. False discovery rates were controlled at 1% at both PSM and protein levels using Philosopher. Only uniquely mapping peptides (those assigned to a single ORF) were retained as evidence of translation.

## Results

### RiboBA method overview

A schematic overview of RiboBA is shown in Fig. 1, with full technical details provided in the “Materials and methods” section and the Supplementary data. In contrast to many existing Ribo-seq tools that assign P-site positions using fixed read-length-dependent offsets, RiboBA explicitly models the protocol-dependent process by which latent ribosomal P-sites give rise to observed RPFs. The generative model captures key technical biases introduced during library construction, including nuclease-specific cleavage bias, 5$^\prime$-end non-templated nucleotide additions, and ligation efficiencies. Concretely, these biases are modeled at specific regions of each footprint: at both the 5$^\prime$ and 3$^\prime$ cut sites, RiboBA models how far the terminus lies from the P-site together with the local sequence at the cut site (a cut-site base preference that is pronounced for MNase but negligible for RNase I and P1); at the 5$^\prime$ end it additionally models non-templated nucleotides added during reverse transcription; and at both termini it models the sequence-dependent efficiency with which the terminal 3-mers are ligated. Because each of these effects is tied to a defined part of the footprint, RiboBA can decompose an observed fragment back to the latent P-site that most likely generated it. Given the estimated parameters, RiboBA assigns each footprint a posterior over candidate P-sites based on its geometry. These weights are used to reassign frame-ambiguous signals, recovering P-site occupancy profiles with clear 3-nt periodicity. The footprint regions modeled by RiboBA and the posterior P-site inference procedure are illustrated in Supplementary Fig. S1, with full technical details in the “Materials and methods” section. The resulting profiles are summarized into CDS-derived features that feed supervised machine-learning models for translated ORF detection. An EM-like alternating optimization scheme, combined with sparse matrix operations, accelerates P-site inference and feature extraction, enabling efficient analysis of large Ribo-seq data sets on standard workstations. Despite the increased model complexity, RiboBA does not impose prohibitive computational overhead, and in our benchmarks, it can achieve runtimes comparable to or faster than those of other widely used ORF-calling tools. Consistent with the outputs summarized in Fig. 1, a single RiboBA run reports protocol-bias diagnostics, identified ncORFs and their biotype composition, and bias-adjusted codon-level occupancy profiles; a complete set of these user-facing outputs for a representative human MNase library is shown in Supplementary Fig. S2.

**Figure 1.**
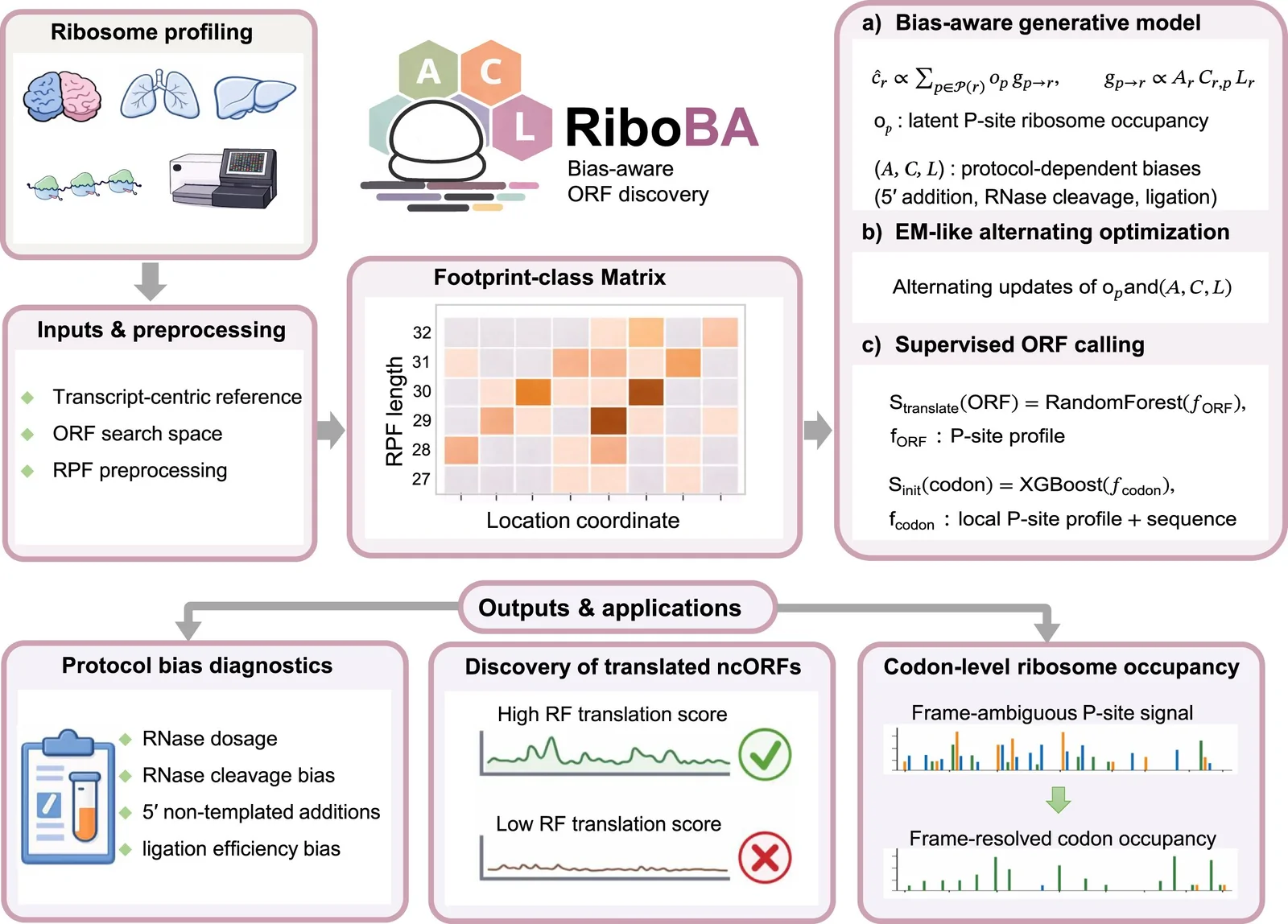
Schematic overview of the RiboBA framework. RiboBA is a bias-aware probabilistic framework for robust ORF identification from Ribo-seq data. The pipeline begins with preprocessing and aligning RPFs to a transcript-level reference to define the candidate ORF search space. A generative module then employs an EM-like optimization procedure to jointly infer protocol-induced biases, such as nuclease cleavage and ligation efficiency, alongside codon-level ribosome occupancy. These bias-adjusted profiles are subsequently utilized by a supervised module to score translated ORFs and initiation sites. The final outputs include protocol-bias diagnostics, identified ncORFs, and adjusted ribosome occupancy profiles for downstream functional analyses.

### Simulation-based validation of bias recovery and P-site inference

As ground truth for bias parameters is unavailable in empirical Ribo-seq data, we used our parameter-recovery simulations to assess the accuracy of RiboBA’s bias-parameter recovery. We generated synthetic libraries across six protocol configurations (see the “Materials and methods” section) using a fixed ribosome occupancy profile. Across all conditions, RiboBA accurately recovered the ground truth bias parameters (Supplementary Fig. S3). Specifically, cleavage profiles for both termini were consistently inferred across RNase I (high- and low-dosage), MNase, and nuclease P1 regimes, with estimated probability curves closely matching the simulated truth (Supplementary Fig. S3A).

Similarly, sequence-dependent ligation preferences were faithfully recovered: estimated 3-mer ligation efficiencies at the 5$^\prime$ and 3$^\prime$ termini showed strong Pearson correlations with the ground truth (average *r ≈ 0.92*; Supplementary Fig. S3B and C). RiboBA also accurately inferred the base-specific cleavage preferences of MNase (Supplementary Fig. S3D) and the probabilities of 5$^\prime$ additions (Supplementary Fig. S3E). Together, these results demonstrate that our generative model robustly infers protocol-induced bias parameters across diverse settings, providing a solid basis for subsequent bias-aware P-site inference and ncORF identification.

We next directly evaluated P-site assignment accuracy on simulated Ribo-seq data with known P-site positions (three independent simulation replicates). The magnitudes below reflect these dataset-specific simulation settings. Compared with the fixed length-specific offsets assigned by riboWaltz, a widely used tool for P-site offset estimation [23], RiboBA assigned a substantially larger fraction of reads to their exact correct nucleotide positions across all three simulated nuclease conditions [RiboBA versus riboWaltz: 73.7% versus 22.7% for RNase I (*+51.0* pp), 22.1% versus 8.7% for MNase (*+13.4* pp), and 39.5% versus 11.9% for nuclease P1 (*+27.6* pp); Supplementary Fig. S4B] and produced P-site metagene profiles around the start and stop codons with more clearly resolved 3-nucleotide periodicity (Supplementary Fig. S4A). Notably, on the MNase data the two tools’ metagene periodicity looks similar; this apparent similarity reflects the intrinsic 3-nucleotide structure of the CDS sequence rather than accurate P-site recovery by riboWaltz, whose much lower per-read accuracy on these data is evident in Supplementary Fig. S4B. At the codon level, RiboBA likewise assigned more reads to their correct codon positions [RiboBA versus riboWaltz: 96.0% versus 78.0% for RNase I (*+18.0* pp), 68.8% versus 29.1% for MNase (*+39.7* pp), and 77.6% versus 38.4% for nuclease P1 (*+39.2* pp); Supplementary Fig. S5A] and more accurately reconstructed true codon-level P-site abundance profiles (Supplementary Fig. S5B), with riboWaltz systematically underestimating true per-codon counts and RiboBA showing no such bias. To examine how protocol-induced biases alter the underlying offset distributions, within each nuclease condition we stratified simulated reads by read length and 5$^\prime$-addition status. These analyses showed that the offset varies substantially not only across read classes but also among reads of the same length, and that RiboBA’s posterior offset distributions capture this within-length heterogeneity, whereas a fixed-offset approach represents only a single length-specific point estimate (Supplementary Fig. S6). At the level of individual reads, RiboBA’s cleavage component alone—the read’s own terminal-sequence evidence, before it borrows the genome-wide P-site abundance prior—yields a broad, often misaligned offset estimate; combining this cleavage evidence with the P-site abundance prior sharpens RiboBA’s full posterior onto the true per-read offset across all three nucleases (Supplementary Fig. S7).

### Simulation-based benchmarking of ORF detection

We next evaluated whether RiboBA improves the identification of translated ORFs relative to existing methods using two simulation frameworks designed to assess detection accuracy under distinct protocol regimes.

The first benchmark, based on positional jittering, evaluated whether each method could distinguish translated CDSs from frame-disrupted signals, using a strategy similar to RiboCode [20]. Positive sets comprised 1000 genes with CDS-aligned reads derived from empirical human Ribo-seq datasets (RNase I, MNase, or nuclease P1). Negative sets consisted of a separate set of 1000 genes from the same datasets, where reads were subjected to positional jittering. This *±*3-nt perturbation disrupts 3-nt periodicity while preserving the overall read distribution, providing a stringent benchmark for evaluating the translation specificity of identified ORFs. Across all three nuclease protocols, RiboBA consistently achieved the highest AUROC and AUPRC values (Supplementary Fig. S8), outperforming RiboCode, PRICE, RibORF, and RiboTISH. ORF-RATER was excluded from this benchmark, as its supervised learning framework is not compatible with the structure of this simulation. These findings demonstrate that explicitly accounting for protocol-induced biases improves the robustness of CDS-level ORF identification under localized frame-disrupting perturbations.

The second benchmark, our synthetic ncORF detection framework, evaluated performance in identifying translated ncORFs under realistic protocol-induced biases. Positive sets comprised 250 translated ncORFs per protocol, simulated from CDS-derived P-site occupancy profiles and empirical bias parameters. Negative sets consisted of an equal number of non-translated ncORFs, generated using identical occupancy profiles but with positional jittering applied to P-site coordinates prior to footprint simulation. This approach ensures that 3-nt periodicity is disrupted while preserving global read distribution and protocol-induced biases, providing a rigorous test for the identification of non-canonical translation. We benchmarked each tool across simulated datasets spanning RNase I, P1, MNase, RNase I with 5$^\prime$ additions, and RNase I with ligation bias. RiboBA consistently achieved the highest AUROC and AUPRC values (Fig. 2A and Supplementary Fig. S9A). Beyond accuracy, RiboBA exhibited the broadest coverage, effectively identifying nearly the entire positive and negative sets (Fig. 2B and Supplementary Fig. S9B). Among the alternative methods, PRICE achieved relatively high classification accuracy, second only to RiboBA, yet exhibited consistently limited coverage across protocols. RiboTISH provided comparatively broad coverage; however, its classification accuracy declined markedly under P1 and MNase regimes. RiboCode displayed a more balanced trade-off between accuracy and coverage overall, but experienced pronounced reductions in both metrics under MNase digestion. In contrast, RibORF and ORF-RATER showed consistently lower accuracy and more restricted coverage across all tested protocol conditions.

**Figure 2.**
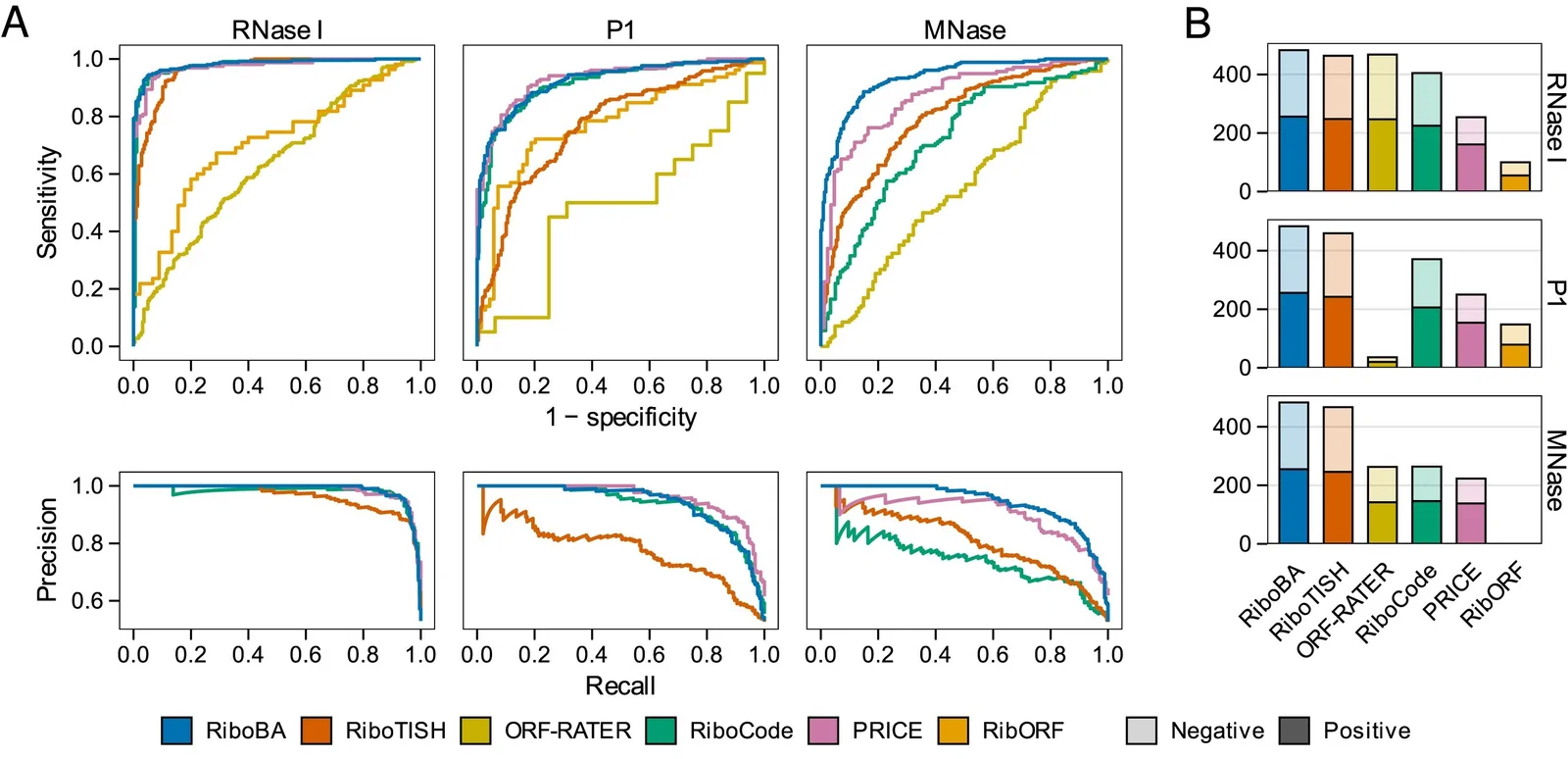
Benchmarking ncORF identification on simulated Ribo-seq datasets. Synthetic Ribo-seq datasets were generated under three nuclease conditions using empirical parameters from HEK293/HEK293T studies (RNase I, P1 and MNase). These datasets were used to evaluate the identification of translated ncORFs across diverse protocols. (**A**) Receiver operating characteristic (ROC) and PR curves comparing RiboBA with PRICE, RiboCode, RibORF, RiboTISH, and ORF-RATER. Performance was assessed based on the method-specific identified ncORFs in each condition. (**B**) Total counts of identified positive and negative ncORFs across the three nuclease regimes.

This combination of high accuracy and broad detection coverage demonstrates that RiboBA provides a more versatile and generalizable framework for the identification of translated ncORFs across diverse experimental conditions. Despite its sophisticated modeling, RiboBA remains computationally efficient: in the runtime benchmark on a representative human Ribo-seq dataset (Lucas RNase I, HEK293T), the total CPU time for genome preparation, sample preprocessing, and ORF inference was 1.9 core-hours for RiboBA, compared with 2.7 core-hours for PRICE and 5.4–30.0 core-hours for the other competing tools (Supplementary Fig. S10).

### Protocol-induced bias in empirical datasets

We next evaluated whether the footprint-generation parameters inferred by RiboBA from empirical Ribo-seq datasets reflect biologically meaningful, protocol-induced biases. To enable cross-protocol comparisons while mitigating biological variability, we curated nine human HEK293/HEK293T Ribo-seq libraries representing diverse library construction protocols and known sources of technical bias.

Using this curated dataset, we first assessed the internal consistency of the protocol-induced parameters inferred by RiboBA within each library. Parameter estimates were independently computed from two random subsets of reads per library across 10 replicate splits. The inferred ribosome protection profiles and 3-mer ligation efficiencies were highly consistent between splits, with Pearson correlation coefficients exceeding 0.99 (Supplementary Fig. S11A and B). Similarly, MNase-specific base preferences and 5$^\prime$ non-templated addition probabilities were highly reproducible (Supplementary Fig. S11C and D), confirming the robustness of the inferred protocol-induced bias parameters.

With robust parameter inference confirmed across replicates, we next evaluated whether the inferred biases reflect biologically meaningful, protocol-induced features of ribosome footprints. We analyzed ribosome protection profiles flanking the P-site across nine protocols (Fig. 3A). RNase I libraries generally exhibited narrow protection windows and steep gradients, consistent with shorter RPFs and more pronounced 3-nt periodicity. However, notable variability was observed across RNase I datasets. For instance, the Calviello library displayed sharp transitions in its protection profile, which were associated with strong periodicity. A similar trend was observed in the Martinez libraries: increasing nuclease concentrations resulted in progressively steeper protection profiles and stronger periodicity, further supporting this association. In contrast, the Lucas replicates yielded highly concordant protection profiles, underscoring the precision and biological fidelity of RiboBA-inferred parameters. Among non-RNase I protocols, MNase libraries showed broad, flat protection profiles, particularly at the 5$^\prime$ end, consistent with prior findings that 3$^\prime$-end-based offsets more reliably capture periodicity under such conditions [41]. P1 libraries exhibited intermediate steepness between RNase I and MNase, reflecting moderate 3-nt periodicity.

**Figure 3.**
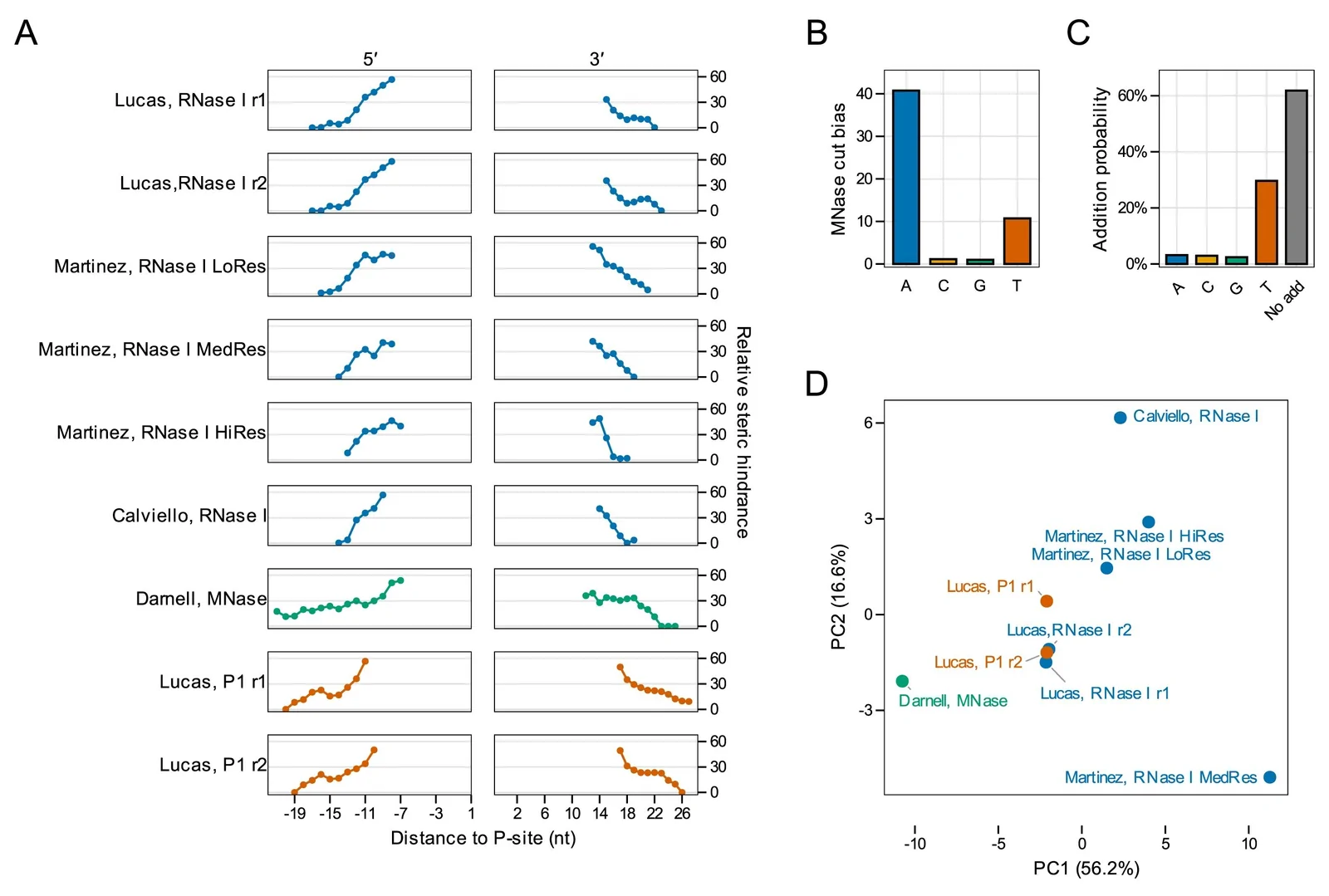
Protocol-induced bias parameters inferred by RiboBA from nine empirical HEK293/HEK293T Ribo-seq datasets. (**A**) Ribosome protection effects represented as terminus-specific relative steric-hindrance profiles flanking the P-site for 5$^\prime$ and 3$^\prime$ read ends; colors indicate the ribonuclease (RNase I, MNase, or P1), and higher values indicate increased resistance to nuclease cleavage. (**B**) MNase-specific cleavage bias in the Darnell dataset, shown as relative base cleavability across A/C/G/T, where larger values indicate higher cleavage susceptibility. (**C**) Estimated ${5^\prime}$ non-templated nucleotide addition probabilities for the Darnell dataset. (**D**) Principal component analysis (PCA) of inferred 3-mer ligation efficiencies at the $3^\prime$ end across the nine datasets; points represent individual libraries and are colored by ribonuclease type.

Next, we examined base-specific nuclease preferences. Biochemical studies have established that MNase preferentially cleaves A/T-rich regions over G/C-rich sites, with cleavage rates differing by up to 30-fold [32]. RiboBA accurately recapitulated this signature, inferring MNase cleavage rates ~40-fold and 10-fold higher for A and T, respectively, compared to C/G in the Darnell dataset (Fig. 3B). In contrast, RNase I and P1 libraries exhibited minimal base-specific preferences (Supplementary Fig. S12A), consistent with the known lower sequence specificity of these enzymes. These results demonstrate that RiboBA faithfully recovers nuclease-induced sequence biases from empirical Ribo-seq data.

We further examined 5$^\prime$ non-templated nucleotide additions. RiboBA estimated low 5$^\prime$-addition probabilities in most RNase I and P1 libraries, but detected markedly elevated levels in the Darnell MNase datasets (Fig. 3C). To assess ligation efficiency bias, we summarized the inferred 3-mer ligation efficiencies at both termini and projected all libraries into a low-dimensional space via principal component analysis (PCA; Fig. 3D and Supplementary Fig. S12B and C). Samples from the same study clustered tightly, while remaining clearly separated from other datasets. This pattern indicates that the inferred ligation parameters capture reproducible biochemical signatures—reflecting the inherent sequence preferences of ligases and adapters—rather than stochastic technical noise.

At the P-site assignment level, and consistent with the simulation results above, RiboBA’s P-site metagene profiles around the start and stop codons showed sharper 3-nucleotide periodicity than the fixed-offset riboWaltz procedure (Supplementary Fig. S13A), and a higher CDS frame-0 P-site fraction (Supplementary Fig. S13B), across both human and *Drosophila* empirical Ribo-seq libraries.

Together, these results demonstrate that RiboBA enables precise and reproducible inference of protocol-induced biases and, on that basis, more accurate P-site assignment across diverse empirical datasets. The inferred parameters may therefore serve as diagnostic metrics and design guides for optimizing future Ribo-seq experiments.

### Benchmarking ncORF identification on empirical Ribo-seq datasets

Having established that RiboBA accurately recovers protocol-induced biases and assigns P-sites accurately, we next evaluated whether this modeling improves the identification of ncORFs in practice. We compared RiboBA with five widely used tools across nine human HEK293/HEK293T Ribo-seq libraries generated using RNase I, MNase, or nuclease P1 protocols.

We first assessed the ORF biotype composition identified by each method across the nine human libraries (Supplementary Fig. S14). As expected, RNase I libraries—which typically exhibit stronger 3-nt periodicity—supported more translated canonical ORFs than MNase or P1 libraries. However, the degree of this protocol dependence varied substantially among tools (Supplementary Fig. S14A). For the five alternative methods, translated canonical ORF counts fluctuated substantially across protocols, ranging from ~1000 to 16 000. This variability suggests that canonical ORF identification by these tools is strongly sensitive to protocol-dependent signal quality. In contrast, RiboBA yielded a more stable identification of 11 000–12 000 canonical ORFs across all protocols. An analogous pattern was observed for ncORFs (Supplementary Fig. S14B), where RiboBA maintained robust identification of uORFs, uoORFs, intORFs, and lncORFs in MNase and P1 libraries, as well as in RNase I datasets with attenuated periodicity. Conversely, alternative methods frequently showed marked reductions under these biased or lower-signal conditions.

We next evaluated the reproducibility of ncORF identifications across biological replicates. Using the Lucas RNase I and P1 replicate pairs, we quantified concordance for each tool and ncORF biotype via both Jaccard similarity and the absolute number of shared identifications (Fig. 4A). For uORFs and uoORFs, RiboBA achieved high replicate concordance while identifying substantially more candidates than alternative methods, a contrast that became more pronounced under the P1 protocol. For other biotypes, no single method consistently dominated, although ORF-RATER showed superior performance for intORFs in both shared identification count and replicate concordance.

**Figure 4.**
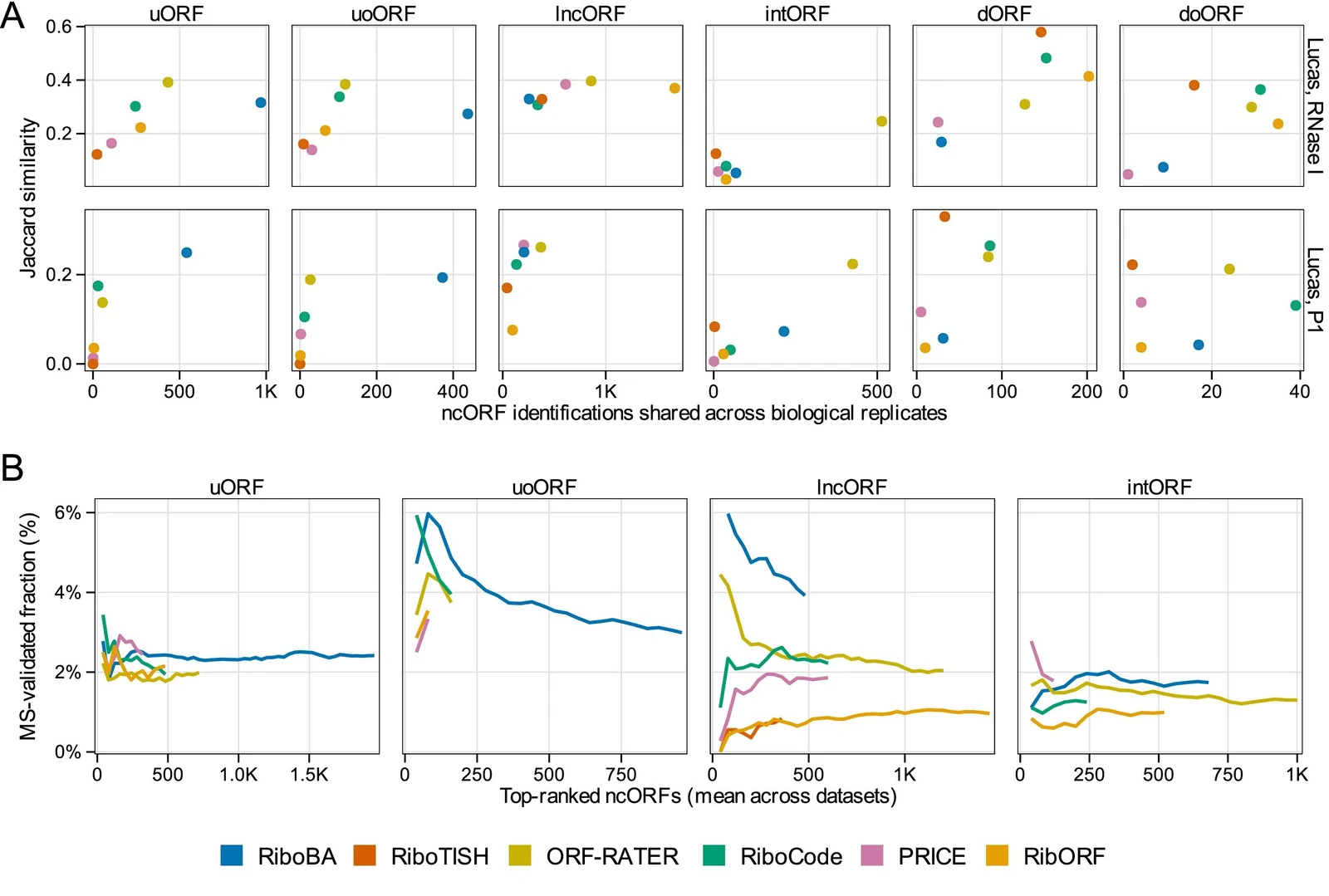
Reproducibility and immunopeptidomics validation of identified ncORFs. ncORFs were identified by RiboBA, PRICE, RiboCode, RibORF, RiboTISH, and ORF-RATER across nine empirical HEK293/HEK293T Ribo-seq datasets. (**A**) Jaccard similarity between biological replicate pairs versus the number of shared ncORF identifications, stratified by ncORF biotype. (**B**) HLA–I immunopeptidomics evaluation: identified ncORFs were ranked by method-specific scores, using the RF translation score for RiboBA, and the cumulative fraction of MS-validated ncORFs was plotted against the number of top-ranked identifications. Data are stratified by biotype (uORF, uoORF, intORF, and lncORF; dORF/doORF are omitted due to sparsity) and averaged across libraries (at least five datasets per data point).

Finally, we sought orthogonal evidence for identified ncORFs using HLA-I immunopeptidomics mass spectrometry—a stringent validation given the short length and low abundance of most ncORF-encoded microproteins [4]. By searching HEK293T MHC class I spectra against custom databases of identified ORFs, we quantified uniquely supported ncORFs after excluding in-frame CDS overlaps (Supplementary Fig. S15). The ncORFs with MS support are summarized in Supplementary Table S2. RiboBA yielded the highest numbers of MS-validated uORFs and uoORFs and ranked among the top methods for intORFs and lncORFs. In contrast, dORFs and doORFs were rarely detected by any method, a result consistent with the high prevalence of non-ribosomal protein-protected fragments in 3$^\prime$ untranslated regions [12].

To evaluate score-based prioritization, we ranked identified ncORFs by their translation scores and assessed MS-support rates across score deciles (Fig. 4B; dORFs and doORFs were excluded due to sparsity). Although overall validation rates were ~2%, distinct enrichment patterns emerged. For uORFs and intORFs, the frequency of MS support remained relatively stable across score ranks; nonetheless, RiboBA identified the largest cohort of MS-validated candidates while maintaining support rates comparable to or higher than those of alternative tools. Conversely, for uoORFs and lncORFs, high translation scores strongly correlated with MS-validated events. In these categories, top-ranked RiboBA identifications achieved validation rates of ~6%, outperforming all other methods in both absolute counts and support density for uoORFs. Notably, while RiboBA identified fewer lncORFs than ORF-RATER or RiboTISH, its validation rate was markedly higher (*∼*4% versus *∼*1%–2%), suggesting higher specificity in identifying bona fide non-canonical translation. In paired library-level tests, RiboBA’s area under the Fig. 4B MS-validation curve (a larger area indicating more MS-validated ncORFs recovered at matched ranking depth) was significantly greater than that of the competing tools in most biotype–tool comparisons—including all comparisons for uORFs and uoORFs—across the human Ribo-seq libraries (one-sided paired Wilcoxon signed-rank tests, BH-corrected; *q< 0.05*; Supplementary Fig. S16).

In summary, our multi-protocol evaluations and orthogonal immunopeptidomics evidence demonstrate the superior performance of RiboBA in identifying bona fide translation events. While alternative tools exhibit protocol-dependent fluctuations in sensitivity, RiboBA maintains stable and robust ncORF identifications even when 3-nt periodicity is attenuated, such as in MNase or P1 libraries. Beyond this robustness, RiboBA identifications of uORFs and uoORFs show higher reproducibility across biological replicates than existing methods. Furthermore, high-scoring RiboBA candidates are significantly enriched for MS-validated peptides, particularly within the uoORF and lncORF classes, where they achieve superior validation rates of ~6%. Collectively, these findings establish RiboBA as a reliable framework for ncORF identification that remains consistent across diverse experimental protocols.

### Case study: ncORF identification in *Drosophila melanogaster*

As a case study, we applied RiboBA to *Drosophila melanogaster*, a system inherently challenging for standard Ribo-seq protocols. Unlike human or yeast, *Drosophila* ribosomes possess an open architecture prone to RNase I-induced disassembly regardless of nuclease dosage [37]. This structural vulnerability necessitates alternative nucleases such as MNase to preserve ribosomal integrity; however, MNase introduces pronounced sequence-dependent cleavage biases that complicate ncORF identification [30]. To evaluate RiboBA’s robustness under these conditions, we analyzed three empirical MNase-based *Drosophila* datasets (Dunn, Greenblatt, and Zhang; Supplementary Table S3).

RiboBA consistently identified a robust set of ncORFs across the three *Drosophila* datasets. Each dataset yielded on the order of several thousand ncORFs spanning diverse biotypes, predominantly uORFs and intORFs, with smaller but consistent contributions from uoORFs, dORFs, doORFs, and lncORFs, and these biotype distributions and initiation patterns were reproducible across datasets (Supplementary Fig. S17A and B). Although ATG was the predominant start codon, near-cognate initiators contributed a consistent fraction across all categories. Comparing ncORFs across the three datasets by shared stop-site position showed that a subset was detected in more than one dataset (Supplementary Fig. S17C). Given that functionally relevant ncORFs often exhibit evolutionary constraints, we evaluated whether RiboBA identifications are enriched for conservation signals. We assessed these ncORFs against matched backgrounds using PhyloCSF for codon-level coding potential [40] and phyloP for nucleotide-level constraints [39]. Across all three datasets, RiboBA-identified ncORFs showed a consistent shift toward higher PhyloCSF scores relative to matched background ORFs that was statistically significant but modest in magnitude (one-sided Wilcoxon rank-sum test; *P* ranging from $5.15\times 10^{-8}$ to $4.43\times 10^{-3}$ across the three datasets for both overlapping and non-overlapping ncORF groups; Fig. 5A and Supplementary Fig. S18A). A shift that is consistent in direction across three independent datasets and statistically significant indicates a genuine enrichment of coding signal among identified ncORFs relative to matched background. The modest magnitude is expected, as most identified ncORFs are not conserved protein-coding genes and may be functional without encoding a conserved protein, therefore scoring near the non-coding background. Complementary phyloP analysis revealed ncORF subsets with nucleotide-level conservation comparable to annotated CDSs (Supplementary Fig. S18B).

**Figure 5.**
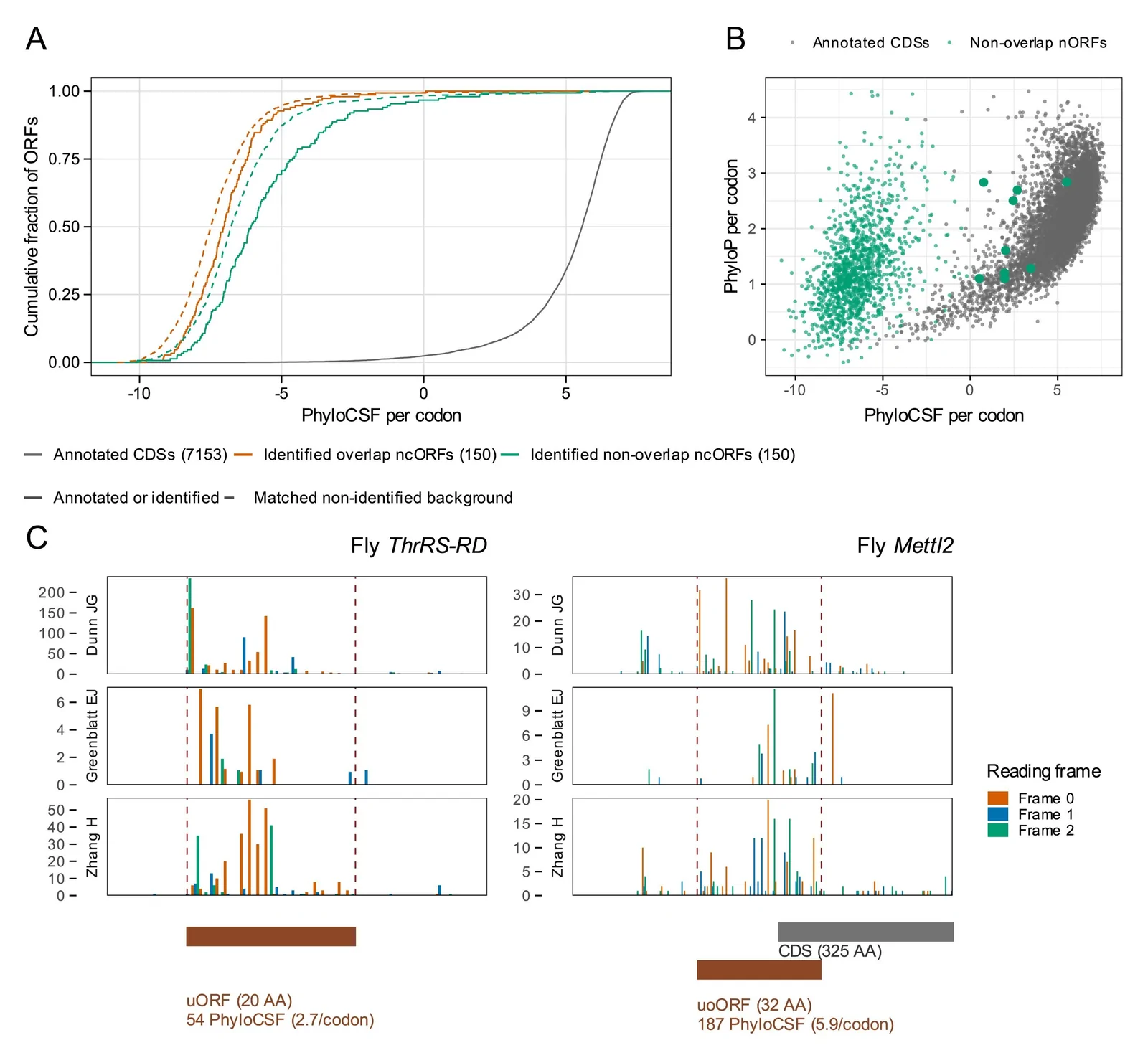
Codon-level conservation and occupancy profiles of RiboBA-identified *Drosophila melanogaster* ncORFs. (**A**) Empirical cumulative distribution functions of per-codon PhyloCSF scores for annotated CDSs and RiboBA-identified ncORFs in the Zhang dataset. ncORFs are grouped into overlapping (uoORF, intORF, or doORF) and non-overlapping (uORF, dORF, or lncORF) categories. Dashed curves represent biotype-matched ORFs from the same transcripts that were not identified as translated. Identified ncORFs were compared with their matched background using one-sided Wilcoxon rank-sum tests (RiboBA-identified *> * matched background); shifts were significant for both groups in this dataset (overlapping, $P=1.83\times 10^{-5}$; non-overlapping, $P=5.15\times 10^{-8}$). (**B**) Joint distribution of per-codon PhyloCSF and phyloP scores for annotated CDSs and non-overlapping ncORFs in the Zhang dataset. (**C**) P-site occupancy profiles for two conserved ncORFs identified across independent datasets (Dunn, Greenblatt, and Zhang): a uORF in *ThrRS* (*ThrRS-RD*) and a uoORF in *Mettl2*. P-site counts are colored by reading frame relative to the highlighted ORF.

To jointly evaluate coding potential and nucleotide-level constraint, we analyzed both PhyloCSF and phyloP scores at the codon level and compared RiboBA-identified ncORFs with annotated CDSs (Fig. 5B and Supplementary Fig. S19). Annotated CDSs clustered distinctly within the high-PhyloCSF/high-phyloP domain, whereas only a small subset of ncORFs overlapped with this region, representing a conserved, coding-like minority. In contrast, a substantial fraction of identified ncORFs exhibited elevated phyloP but negative PhyloCSF scores. This pattern is consistent with conserved *cis*-regulatory roles rather than stable protein-coding functions, a trend particularly prominent among uORFs [10, 11, 42, 43].

Among the high-confidence ncORFs with positive PhyloCSF scores (Supplementary Table S3), RiboBA identified a conserved, translated uORF in *ThrRS* (FBgn0027081) and a uoORF within *Mettl2* (FBgn0035247) across independent datasets (Fig. 5C). Both genes play essential roles in threonine-linked decoding: *ThrRS* encodes the threonyl-tRNA synthetase for charging tRNA${}^{\mathrm{Thr}}$, which also functions as a non-canonical translation initiation factor [44], while *Mettl2* encodes a conserved tRNA methyltransferase that, in mammals, installs m${}^{3}$C at position 32 of tRNA${}^{\mathrm{Thr}}$ isoacceptors [45]. The recurrent identification of upstream translation in both a synthetase and its corresponding tRNA modifier suggests a potential regulatory axis coordinating threonine-specific translational fidelity. In summary, our analysis of *Drosophila* datasets demonstrates that RiboBA effectively overcomes protocol-specific enzymatic biases to identify biologically relevant and evolutionarily conserved ncORFs. These findings highlight the capacity of RiboBA to uncover novel translational control mechanisms in experimentally challenging systems.

## Discussion

Systematic biases introduced during Ribo-seq library preparation can obscure the 3-nt periodicity often used for precise identification of translated ncORFs [46]. To address this challenge, we developed RiboBA, a bias-aware framework that models footprint generation as a probabilistic mapping from latent P-site positions to observed reads and then scores candidate ORFs using bias-adjusted ribosome occupancy profiles. Across simulations and multi-protocol empirical datasets from human and *Drosophila*, RiboBA showed its clearest comparative advantages under technically challenging protocols, particularly in libraries with attenuated periodicity or pronounced protocol-dependent distortion. Rather than relying on strong periodicity as a default assumption, RiboBA explicitly models sources of signal distortion that current ORF callers often treat only implicitly or ignore. Collectively, these results support RiboBA as a robust framework for ncORF identification across heterogeneous Ribo-seq datasets.

The bias profiles inferred by RiboBA represent more than auxiliary parameters for ORF identification; they also provide interpretable diagnostic readouts for experimental evaluation and cross-study comparison. For example, the inferred sharpness of ribosome protection offers a quantitative indicator of nuclease digestion stringency, whereas other bias components highlight distortions introduced during cleavage, ligation, or reverse transcription. Beyond individual experiments, these inferred profiles define sample-specific bias signatures that summarize protocol-dependent variation across datasets. As the field moves toward unified and comprehensive translatome annotation [47, 48], such explicit representations of technical heterogeneity may facilitate more reliable normalization, integration, and comparison of Ribo-seq data generated across laboratories and protocols.

Despite these advances, reliable detection of short and weakly translated ncORFs remains challenging. This limitation reflects not only methodological constraints but also intrinsic properties of Ribo-seq data, as RPFs are short and often unevenly distributed along transcripts. In addition, the current implementation of RiboBA adopts a simplified one-representative-transcript-per-gene strategy, whereas translation frequently involves multiple isoforms with overlapping sequences and substantial alignment ambiguity [49, 50]. This simplification is expected to affect the identification of ncORF biotypes to different extents. uORFs and uoORFs are likely to be most sensitive because their definition depends directly on transcript-specific 5$^\prime$ UTR structures and CDS start sites, whereas dORFs and doORFs may be affected by alternative 3$^\prime$ UTRs and CDS stop sites. intORFs may change overlap status or reading-frame relationship across coding isoforms. lncORFs are less directly affected by protein-coding isoform choice, but they remain sensitive to lncRNA isoform annotation and exon usage. Independently of transcript choice, exact start-codon assignment remains harder for ncORFs with low ribosome occupancy and for ncORFs that overlap annotated CDSs, and such initiation sites should be interpreted with caution even when translation is well supported. Future extensions of RiboBA will therefore focus on integrating signals across multiple Ribo-seq datasets and, ultimately, across multiple transcript isoforms. By moving from single-library inference toward integrative, cross-protocol modeling, we aim to improve the high-resolution annotation of the dark translatome.

## Supplementary Material

gkag969_Supplemental_Files

## Data Availability

The RiboBA package is publicly available at GitHub (https://github.com/Bai-JunYu/RiboBA) and archived at Zenodo (https://doi.org/10.5281/zenodo.19076580) [51]. Scripts used for benchmarking, downstream validation, and figure generation are available in the companion repository RiboBA_analysis (https://github.com/Bai-JunYu/RiboBA_analysis) and archived at Zenodo (https://doi.org/10.5281/zenodo.19076571) [52]. All code is released under the MIT License. All raw and processed data used to generate the main and supplementary figures have been deposited in Zenodo (https://doi.org/10.5281/zenodo.19070762) [53].
