# Supplementary material for: RiboBA: a bias-aware probabilistic framework for robust ORF identification across diverse ribosome profiling protocols
Source: Nucleic Acids Res. 2026 Oct 14;54(19):gkag969. doi: 10.1093/nar/gkag969 (PMC13662281; doi:10.1093/nar/gkag969)
Supplement: Supplementary file 1 [file gkag969_supplemental_files.zip › NAR-01180-Met-K-2026_Supplementary.pdf]

Supplemental Information

RiboBA: a bias-aware probabilistic framework for robust ORF identification across diverse ribosome profiling protocols

Junyu Bai[1], Ruolin Yang[#,1]

[1]College of Life Sciences, Northwest A&F University, Yangling, Shaanxi, 712100 China

# Correspondence to RY (desert.ruolin@gmail.com)

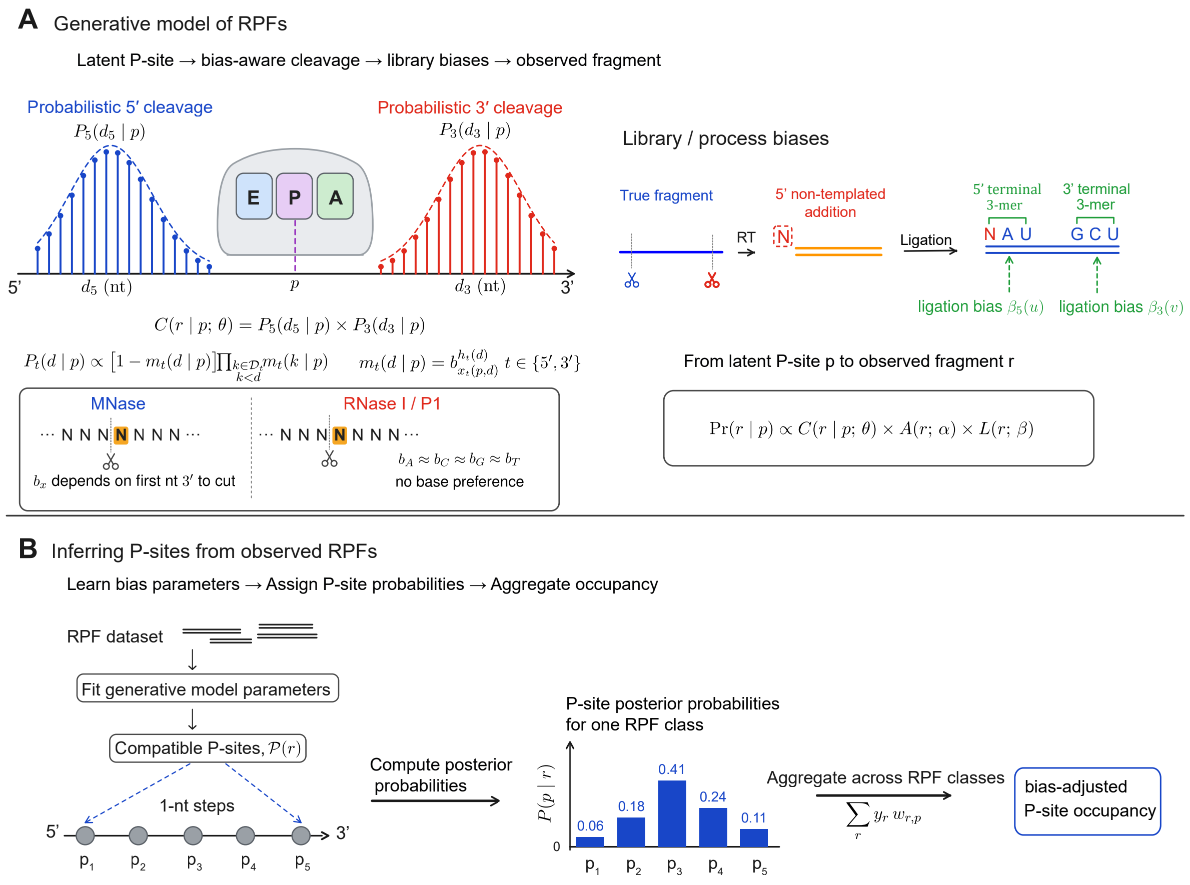

Supplementary Figure S1. RiboBA generative model and P-site inference. (A) Generative model of RPFs. The probability of observing a fragment from a latent P-site factorizes into a nuclease-specific cleavage term, a 5′ non-templated addition term, and a ligation-sequence bias term. The cleavage term models the 5′ and 3′ end-to-P-site distances, with the cut position shaped by the surrounding sequence, whose per-base cleavage preference is marked for MNase but negligible for RNase I and P1. **(B)** P-site inference procedure. RiboBA fits the model by expectation-maximisation, enumerates compatible P-site positions for each RPF class in 1-nt steps, and aggregates read-count-weighted posteriors into bias-adjusted P-site occupancy.

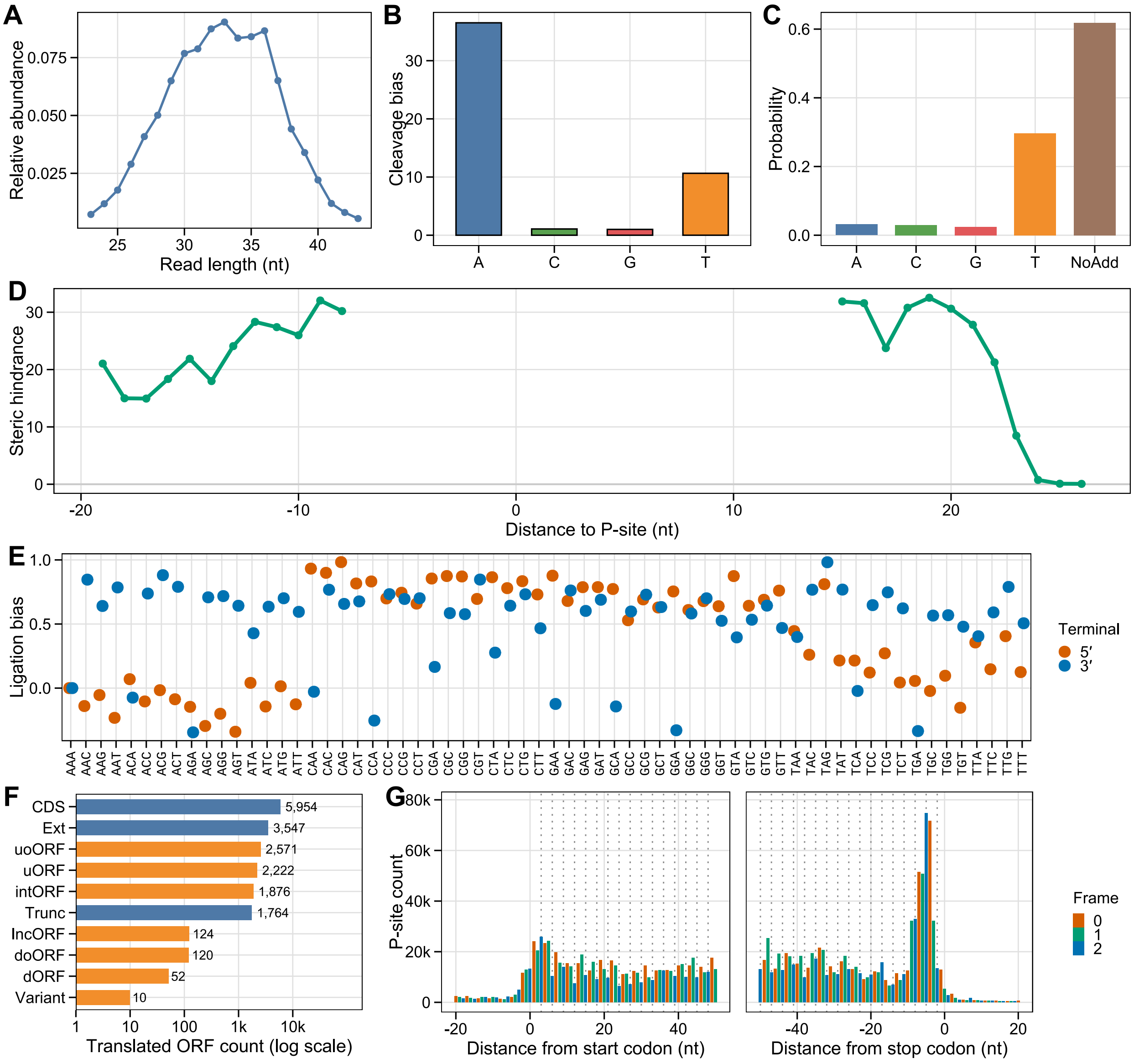

A
Relative abundance
0.075
0.050
0.025
25
30
35
40
Read length (nt)
B
Cleavage bias
30
20
10
0
A
C
G
T
C
Probability
0.6
0.4
0.2
0.0
A
C
G
T
NoAdd
D
Steric hindrance
30
20
10
0
-20
-10
0
10
20
Distance to P-site (nt)
E
Ligation bias
1.0
0.5
0.0
AAA AAC AAG AAT ACA ACC ACG ACT AGA AGC AGG AGT ATA ATC ATG ATT CAA CAC CAG CAT CCA CCC CCG CCT CGA CGC CGG CGT CTA CTC CTG CTT GAA GAC GAG GAT GCA GCC GCG GCT GGA GGC GGG GGT GTA GTC GTG GTT TAA TAC TAG TAT TCA TCC TCG TCT TGA TGC TGG TGT TTA TTC TTG TTT
Terminal
5′
3′
F
CDS 5,954
Ext 3,547
uoORF 2,571
uORF 2,222
intORF 1,876
Trunc 1,764
lncORF 124
doORF 120
dORF 52
Variant 10
1
10
100
1k
10k
Translated ORF count (log scale)
G
P-site count
80k
60k
40k
20k
0
-20
0
20
40
Distance from start codon (nt)
-40
-20
0
20
Distance from stop codon (nt)
Frame
0
1
2

Supplementary Figure S2. RiboBA user-facing output examples for a representative human MNase Ribo-seq library (Darnell *et al.*). **(A)** RPF length distribution estimated by RiboBA. **(B)** MNase cleavage base preference (nucleotide-specific cleavage bias). **(C)** 5′ non-templated nucleotide addition profile; y-axis, probability of 5′ single-nucleotide addition; NoAdd denotes reads with no added nucleotide. **(D)** Steric hindrance profile: the relative steric-hindrance magnitude as a function of distance to the P-site, for the 5′ (negative distances) and 3′ (positive distances) footprint ends. **(E)** Ligation biases for all 64 3-mers at the 5′ and 3′ ends shown as a dot plot; orange dots, 5′ end; blue dots, 3′ end. **(F)** Biotype composition of translated ORFs identified by RiboBA ($\log_{10}$ scale; numbers at each bar give the ORF count). Biotype labels: CDS, annotated coding sequence; Trunc, truncated CDS; Ext, extended CDS; uORF, upstream ORF; dORF, downstream ORF; uoORF, upstream overlapping ORF; doORF, downstream overlapping ORF; intORF, internal ORF overlapping the CDS in a different reading frame; lncORF, long noncoding RNA ORF. **(G)** Bias-adjusted RiboBA P-site occupancy metagene profiles around the start codon (left panel) and stop codon (right panel), coloured by the reading frame of each P-site position relative to the CDS (frame 0, in-frame). Dotted vertical lines indicate 3-nt codon boundaries within the CDS.

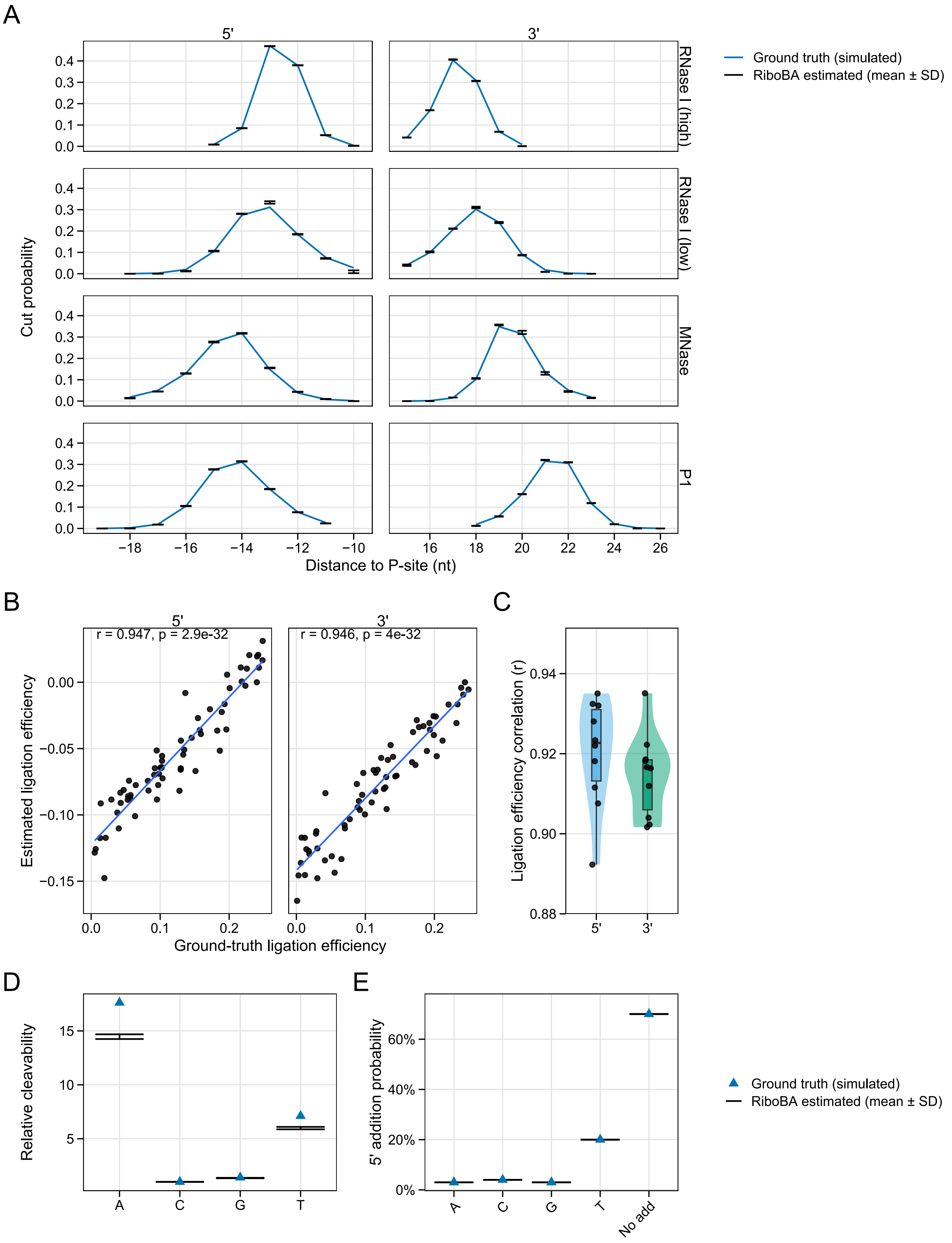
A
5'
3'
RNase I (high)
RNase I (low)
MNase
P1
Cut probability
0.0
0.1
0.2
0.3
0.4
−18
−16
−14
−12
−10
16
18
20
22
24
26
Distance to P-site (nt)
Ground truth (simulated)
RiboBA estimated (mean ± SD)
B
5'
3'
r = 0.947, p = 2.9e-32
r = 0.946, p = 4e-32
Estimated ligation efficiency
0.00
−0.05
−0.10
−0.15
0.0
0.1
0.2
Ground-truth ligation efficiency
C
Ligation efficiency correlation (r)
0.94
0.92
0.90
0.88
5'
3'
D
Relative cleavability
15
10
5
A
C
G
T
E
5' addition probability
60%
40%
20%
0%
A
C
G
T
No add
Ground truth (simulated)
RiboBA estimated (mean ± SD)

Supplementary Figure S3. Recovery of protocol-induced bias parameters in simulations. (A) Terminus-specific cut probability profiles flanking the P-site for RNase I (high/low dosage), MNase, and P1, shown separately for 5′ and 3′ read ends. Coloured lines show the ground-truth (simulated) profiles; black error bars show the RiboBA estimates (mean ± SD across $n = 10$ simulations). **(B–C)** Recovery of ligation bias parameters. **(B)** Correlation between mean estimated and ground-truth 3-mer ligation efficiencies across 10 simulations, with Pearson coefficients indicated for both termini. **(C)** Pearson correlations between estimated and ground-truth ligation parameters across 10 independent simulated libraries. **(D)** MNase-specific cleavage bias shown as relative cleavability across A/C/G/T (triangles, ground truth; horizontal lines, RiboBA estimated; mean ± SD; $n = 10$). **(E)** 5′ non-templated nucleotide addition probabilities (triangles, ground truth; horizontal lines, RiboBA estimated; mean ± SD; $n = 10$).

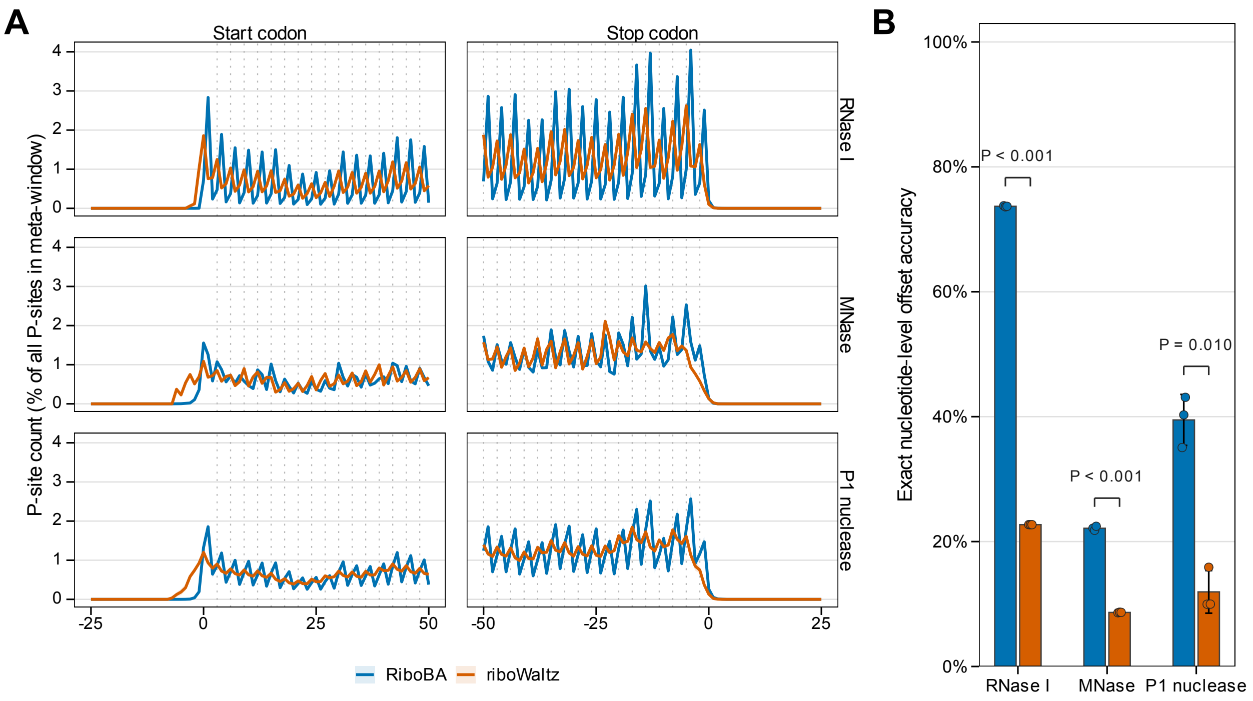

Supplementary Figure S4. Simulated P-site assignment and periodicity validation. Ribo-seq reads were simulated under RNase I, MNase, and P1 nuclease conditions with known P-site positions. **(A)** Meta-profiles of the P-site count at each position, as a percentage of all P-sites in the meta-window, around annotated start and stop codons, averaged across three simulation replicates. Rows show each nuclease condition; dotted gridlines at 3-nt intervals highlight triplet periodicity. **(B)** Exact nucleotide-level offset accuracy, defined as the fraction of reads assigned to the true P-site nucleotide. Bars show means $\pm$ SD across three replicates, with individual replicates as points. P values are from paired t-tests between tools across the three matched replicates ($n = 3$; effect sizes are large relative to the between-replicate SD).

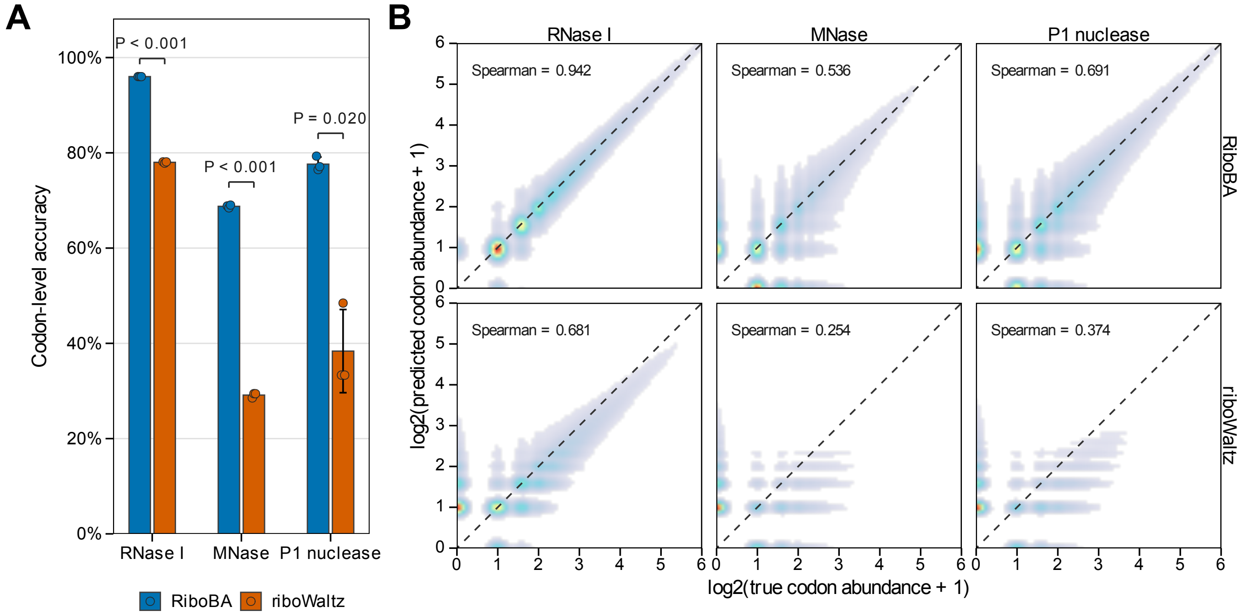

Supplementary Figure S5. Simulated codon-level P-site validation. Codon-level performance was evaluated using the same simulated Ribo-seq datasets with known P-site positions. **(A)** Codon-level assignment accuracy, defined as the fraction of reads whose inferred P-site falls in the correct ribosomal codon. Bars show means $\pm$ SD across three replicates; points show individual replicates. P values are from paired t-tests between tools across the three matched replicates ($n = 3$; effect sizes are large relative to the between-replicate SD). **(B)** Two-dimensional density scatter of true vs. predicted per-codon P-site counts after $\log_2(\text{count} + 1)$ transformation; columns: nuclease condition, rows: tool. Dashed diagonal indicates perfect agreement; Spearman ρ quantifies rank concordance.

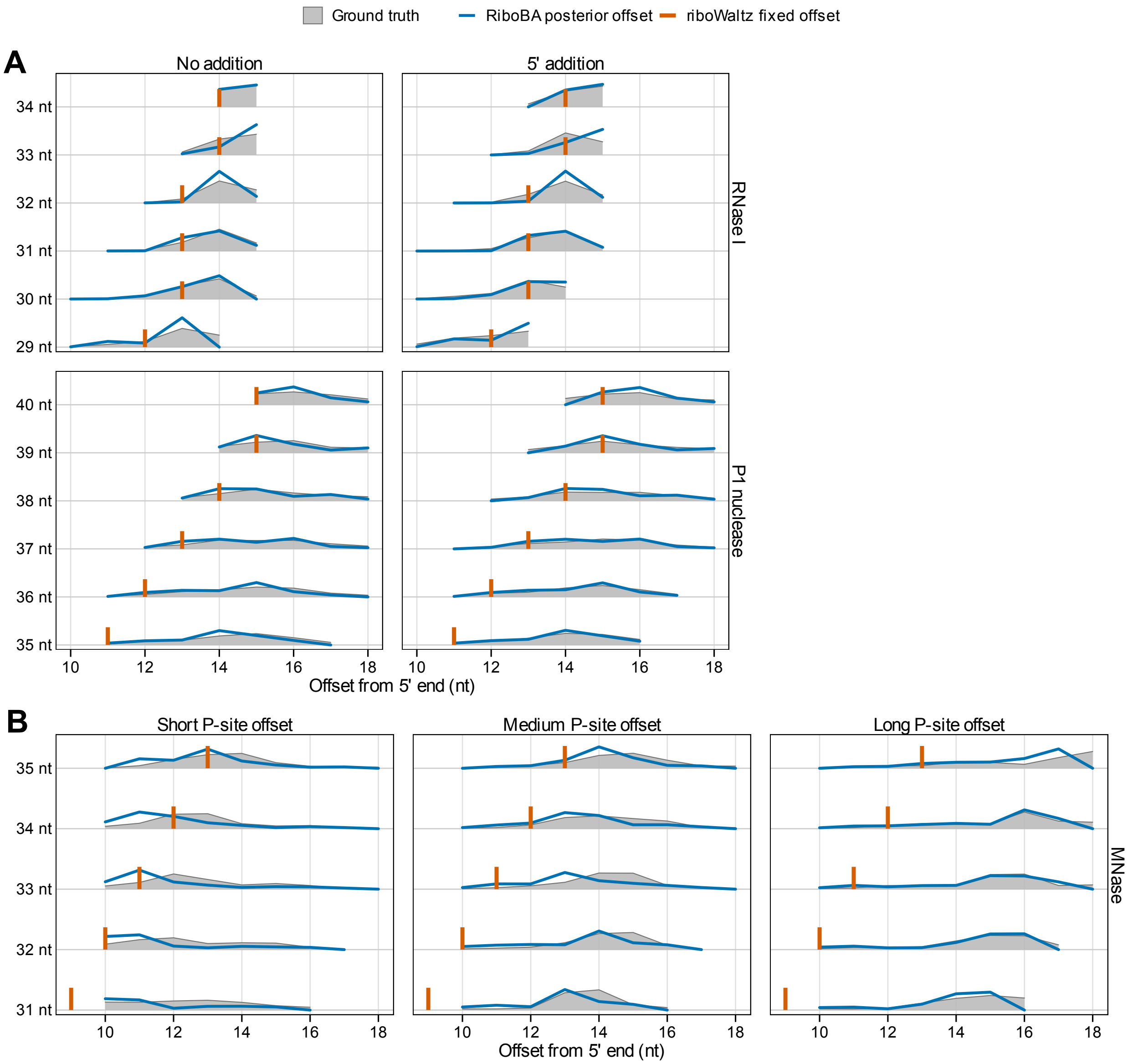

Supplementary Figure S6. Per-read-class P-site offset distributions under three simulated nuclease conditions. P-site offset distributions were estimated from simulated Ribo-seq datasets with known true P-site positions; grey filled areas: ground-truth distributions; blue outlines: RiboBA posterior distributions; orange vertical lines: riboWaltz fixed length-specific offsets. **(A)** RNase I and P1 nuclease simulations, stratified by read length and 5′-addition status (no addition vs. 5′ non-templated addition). **(B)** MNase simulations, stratified by read length. Within each length class, offset distributions vary by sequence context; reads are grouped into short, medium, and long P-site offset to display this heterogeneity.

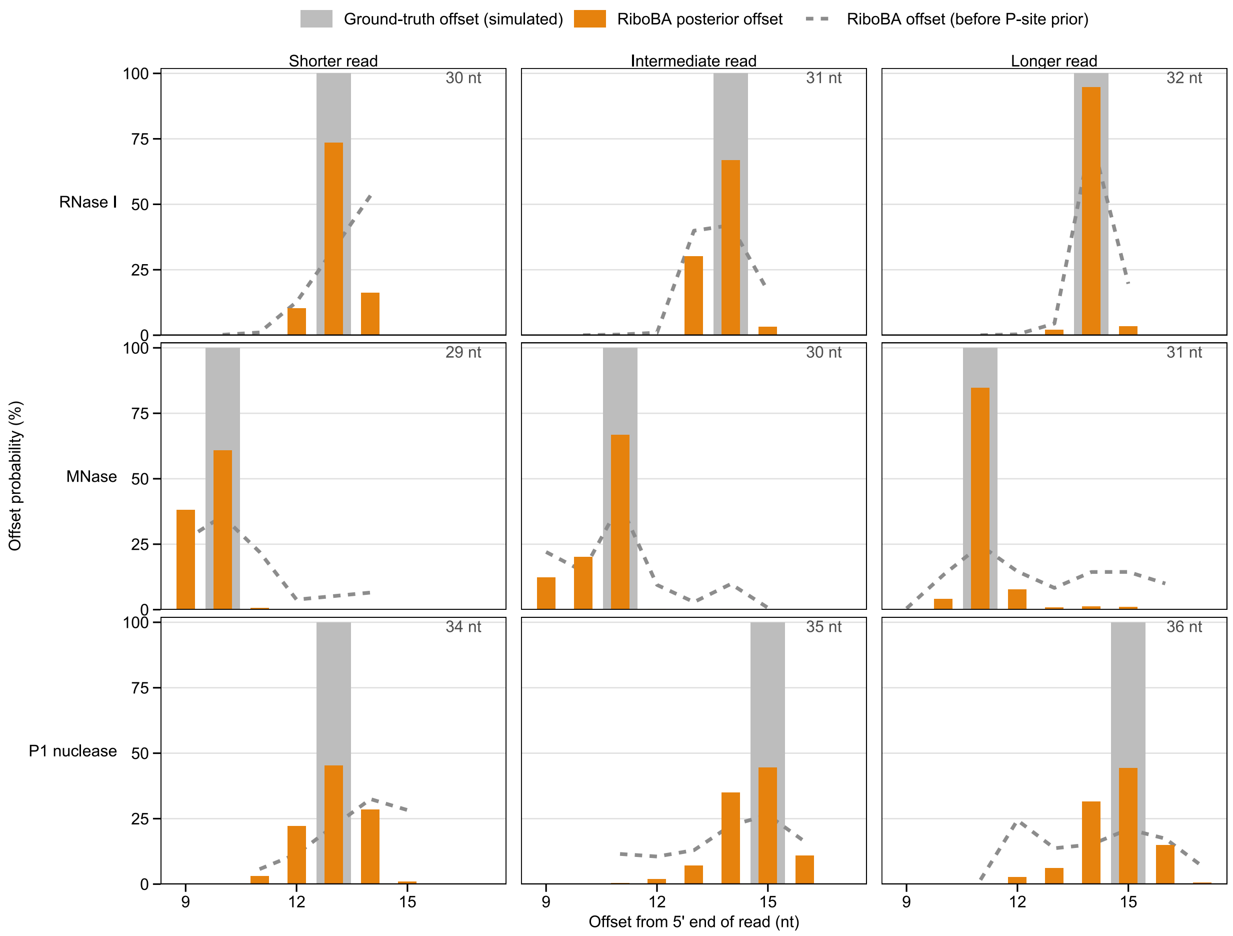

Supplementary Figure S7. Per-read RiboBA offset posterior versus ground truth across three nucleases. For simulated reads with known true P-sites, rows correspond to RNase I, MNase, and nuclease P1; columns show three randomly chosen reads of adjacent read lengths (indicated in each panel), one per length. In each panel: the grey bar marks the ground-truth offset; orange bars, the full RiboBA posterior over candidate offsets; the dashed line, the estimate from the cleavage component alone (before the read borrows the genome-wide P-site abundance prior). The RiboBA posterior tracks the ground-truth offset, whereas the cleavage-only estimate is broader and less well aligned.

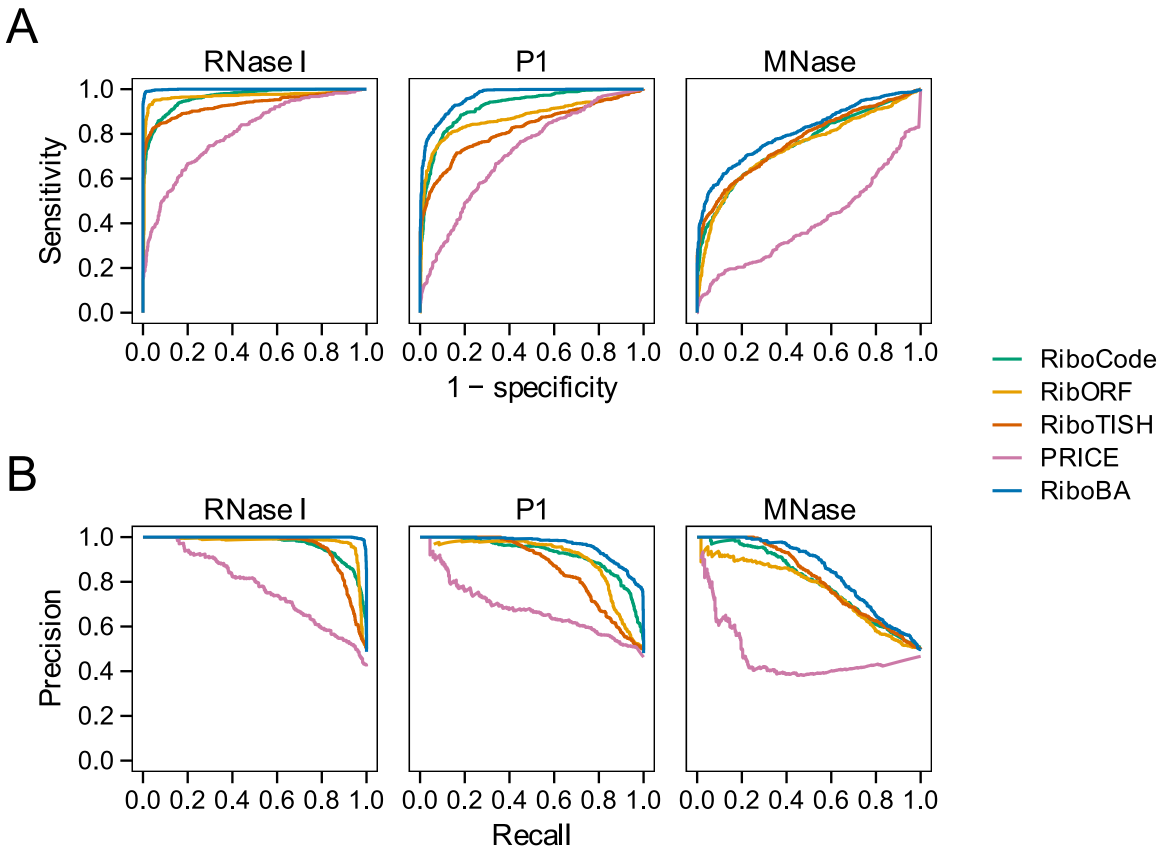

Supplementary Figure S8. Performance benchmarking on CDS-level Ribo-seq simulations. Synthetic datasets were derived from empirical HEK293/HEK293T Ribo-seq experiments (RNase I and P1 from Lucas *et al.*; MNase from Darnell *et al.*). **(A)** Receiver operating characteristic (ROC) curves comparing RiboBA with PRICE, RiboCode, RibORF, and RiboTISH. **(B)** Precision–recall (PR) curves comparing the same five tools. ORF-RATER was excluded from this benchmark because its supervised scoring framework is incompatible with the positional-jittering simulation design.

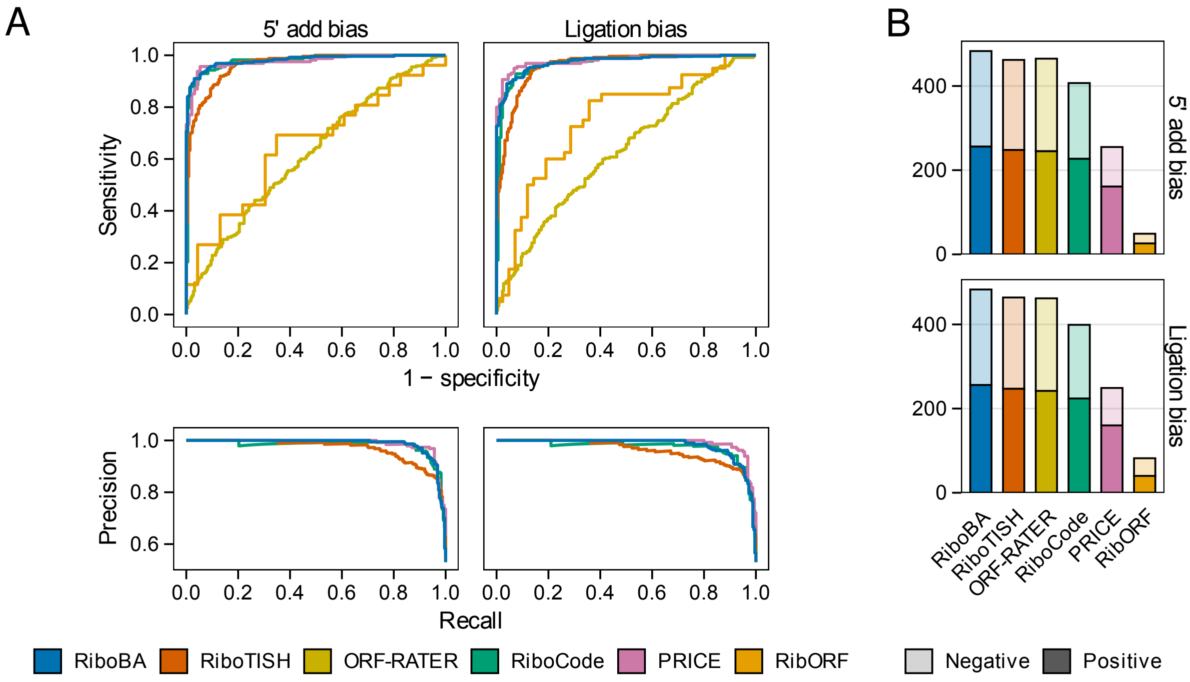

Supplementary Figure S9. Performance of ncORF identification under simulated protocol-induced biases. The benchmarking analysis was performed on synthetic datasets incorporating 5′ non-templated addition or ligation bias (parameters derived from empirical RNase I data, Lucas *et al.*). **(A)** Receiver operating characteristic (ROC) and precision–recall (PR) curves computed from identified positive and negative ncORFs for each method. **(B)** Total counts of identified positive and negative ncORFs across each protocol configuration.

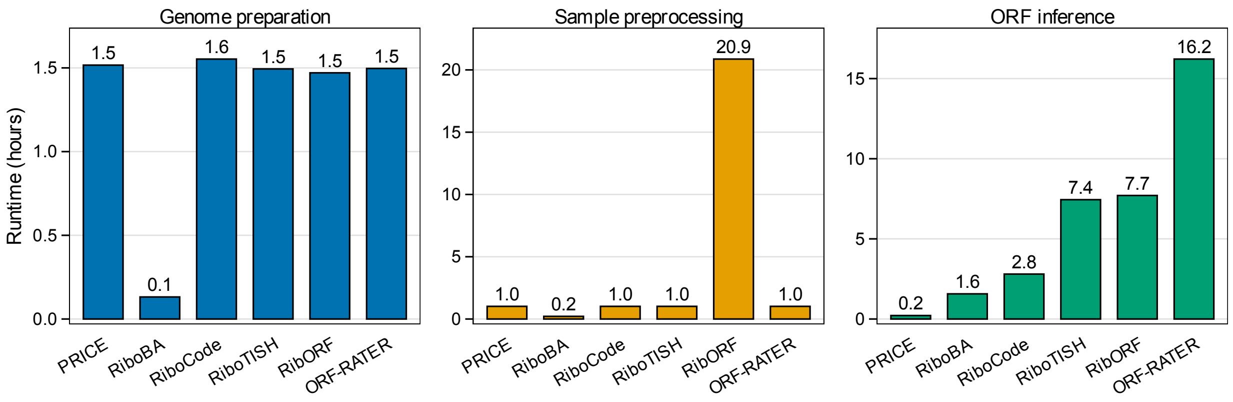

Supplementary Figure S10. Computational efficiency of ncORF identification. Total CPU time (core-hours) required for the identification of translated ncORFs from the Lucas RNase I human HEK293T Ribo-seq dataset, partitioned into genome preparation, sample preprocessing, and ORF-level inference.

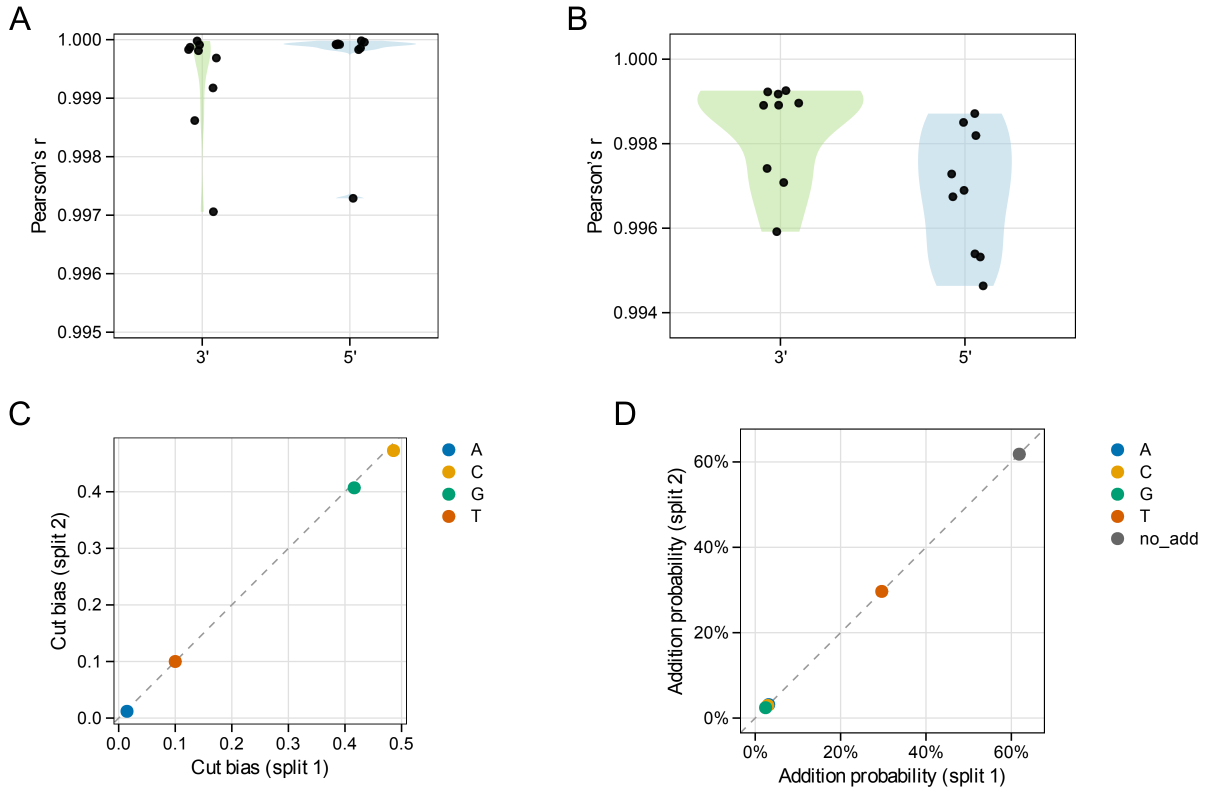

Supplementary Figure S11. Internal consistency of protocol-induced bias parameters inferred by RiboBA from empirical HEK293/HEK293T Ribo-seq datasets. (A–B) Pearson correlation coefficients (r) between two random read partitions for ribosome protection effects **(A)** and 3-mer ligation efficiencies **(B)**, evaluated for $5'$ and $3'$ termini (each point represents one dataset). **(C–D)** Agreement between partitions for MNase cleavage bias **(C)** and $5'$ non-templated addition probabilities **(D)** in the Darnell dataset. In scatter plots, the dashed line indicates $y = x$.

A

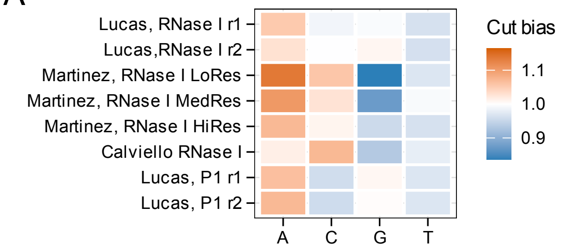

B

Lucas, RNase I r1	1.00	0.99	0.67	0.71	0.39	0.30	0.35	0.81	-0.02
Lucas,RNase I r2	0.99	1.00	0.69	0.73	0.45	0.37	0.41	0.83	0.06
Lucas, P1 r1	0.40	0.34	1.00	0.99	0.39	0.34	0.60	0.57	0.29
Lucas, P1 r2	0.37	0.30	0.96	1.00	0.40	0.39	0.59	0.60	0.28
Martinez, RNase I LoRes	0.59	0.65	0.09	-0.02	1.00	0.86	0.72	0.53	0.19
Martinez, RNase I MedRes	0.48	0.53	0.05	-0.11	0.75	1.00	0.72	0.44	0.16
Martinez, RNase I HiRes	0.27	0.30	0.15	0.03	0.47	0.81	1.00	0.49	0.43
Darnell, MNase	0.13	0.11	-0.05	0.09	-0.24	-0.52	-0.50	1.00	0.05
Calviello, RNase I	0.63	0.66	-0.09	-0.15	0.61	0.69	0.42	-0.19	1.00

C

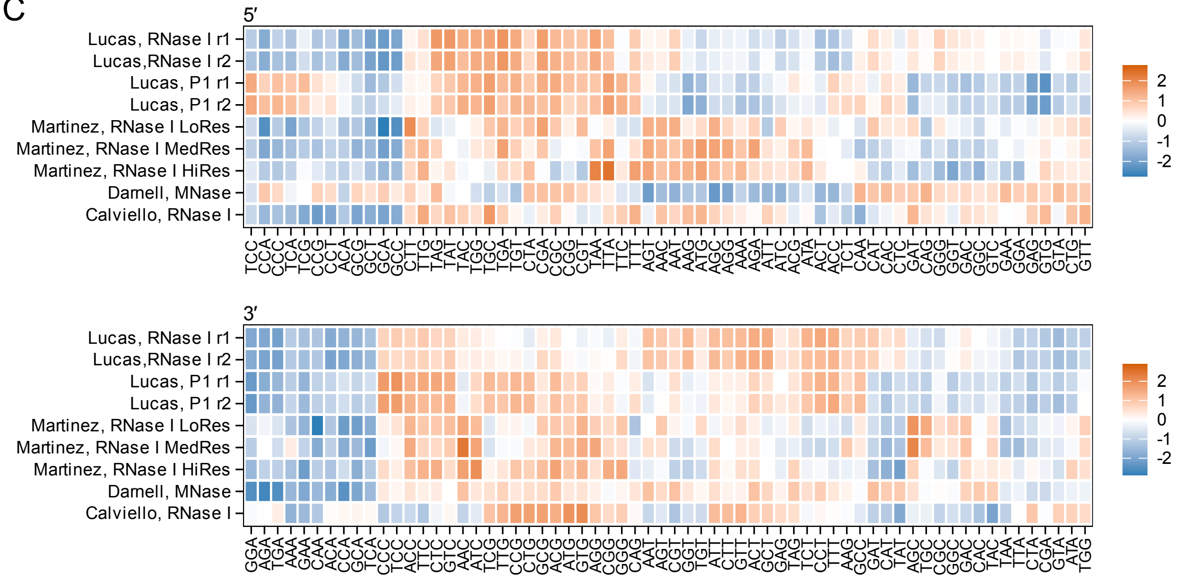

Supplementary Figure S12. Characterization of nuclease cleavage and ligation biases across nine empirical HEK293/HEK293T Ribo-seq datasets. (A) Base-specific nuclease cleavage bias across A/C/G/T shown as relative cleavability for the eight non-MNase datasets. **(B)** Pearson correlation matrix of inferred 3-mer ligation efficiencies across datasets (5′ terminus, lower triangle; 3′ terminus, upper triangle). **(C)** Heatmaps of inferred 3-mer ligation efficiencies for 5′ (top) and 3′ (bottom) termini across all libraries.

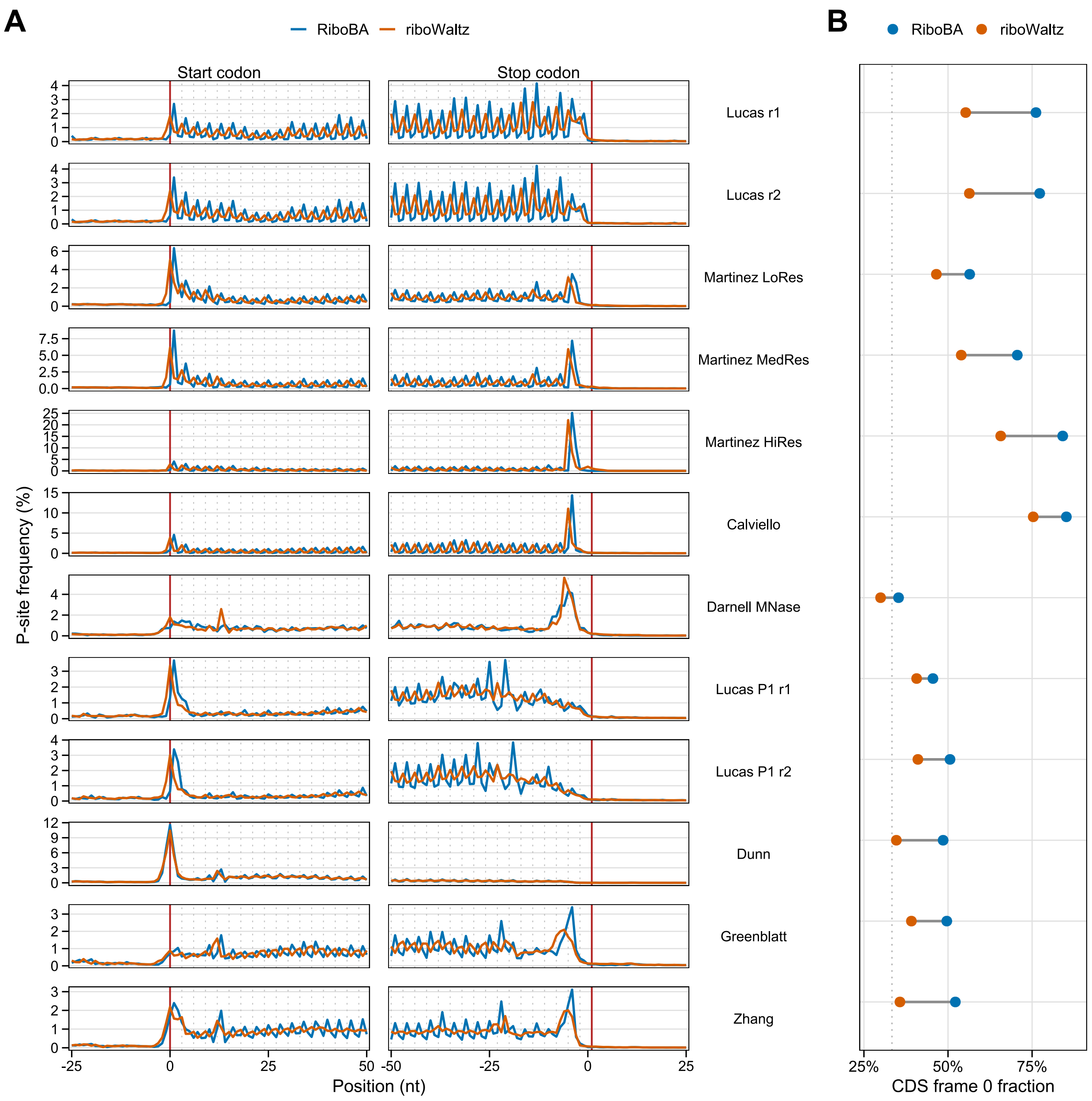

Supplementary Figure S13. Comparison of P-site 3-nt periodicity between RiboBA and riboWaltz across real Ribo-seq libraries. (A) P-site meta-profiles around start and stop codons for twelve Ribo-seq libraries (blue: RiboBA; orange: riboWaltz). Top nine rows: human datasets (RNase I: Lucas r1/r2, Martinez LoRes/MedRes/HiRes, Calviello; MNase: Darnell; P1: Lucas P1 r1/r2). Bottom three rows: *Drosophila melanogaster* datasets (Dunn, Greenblatt, Zhang). **(B)** CDS frame 0 P-site fraction per library for RiboBA (blue) and riboWaltz (orange). Dotted line at 33.3% marks the uniform-distribution baseline.

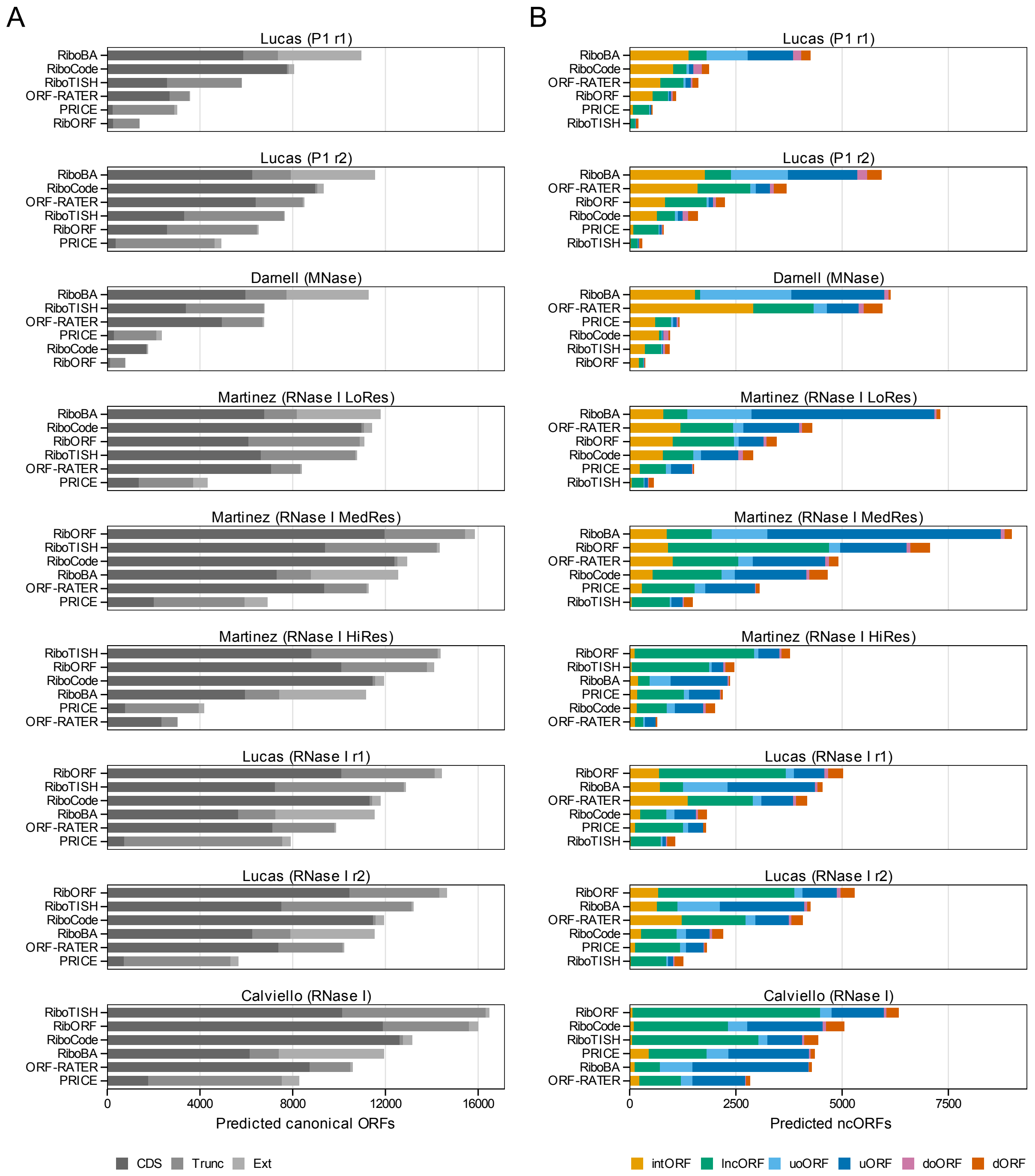
A
B
Lucas (P1 r1)
Lucas (P1 r2)
Darnell (MNase)
Martinez (RNase I LoRes)
Martinez (RNase I MedRes)
Martinez (RNase I HiRes)
Lucas (RNase I r1)
Lucas (RNase I r2)
Calviello (RNase I)
RiboBA
RiboCode
RiboTISH
ORF-RATER
PRICE
RibORF
0
4000
8000
12000
16000
Predicted canonical ORFs
CDS
Trunc
Ext
0
2500
5000
7500
Predicted ncORFs
intORF
lncORF
uoORF
uORF
doORF
dORF

Supplementary Figure S14. Biotype composition of identified ORFs across nine empirical human Ribo-seq datasets. ORFs identified by each method were quantified across nine human Ribo-seq datasets and grouped by biotype. **(A)** Canonical ORFs, including annotated, truncated, and extended CDS regions. **(B)** Non-canonical ORFs (ncORFs), including uORF, uoORF, intORF, dORF, doORF, and lncORFs.

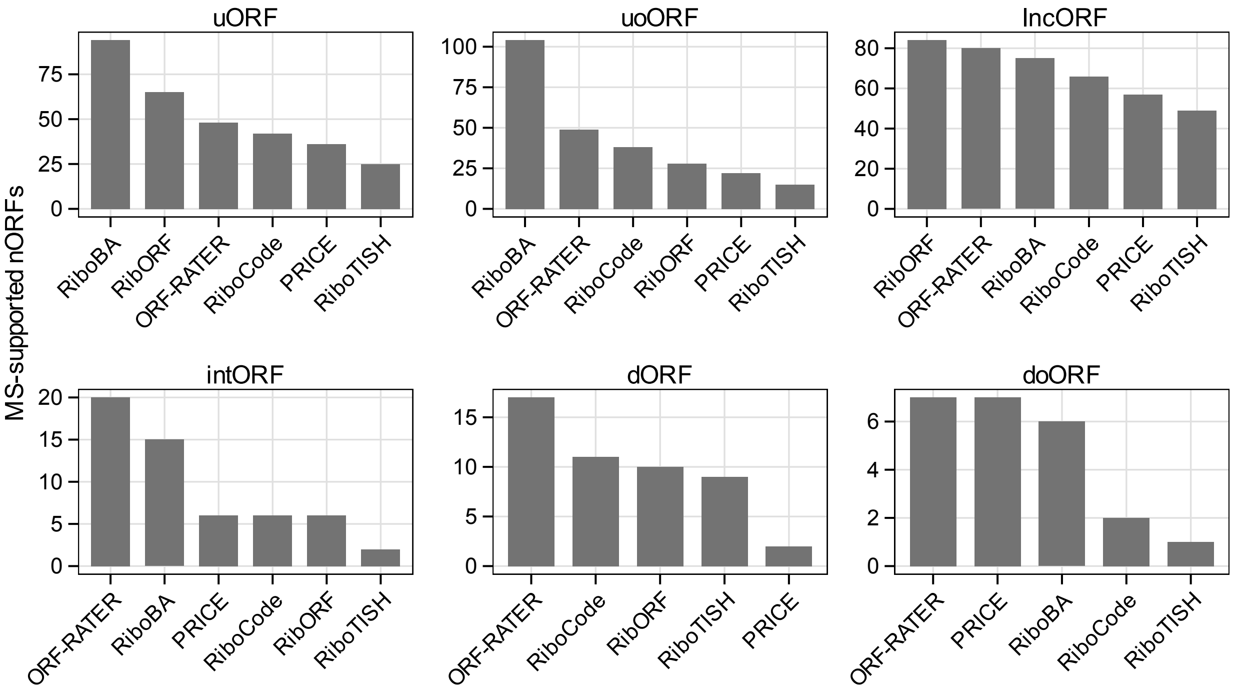

Supplementary Figure S15. MS validation of identified ncORFs across nine empirical HEK293/HEK293T Ribo-seq datasets. Identified ncORFs were evaluated using independent HLA–I immunopeptidomics mass spectrometry evidence. Bars represent the number of uniquely MS-validated ncORFs for each method, stratified by ncORF biotype.

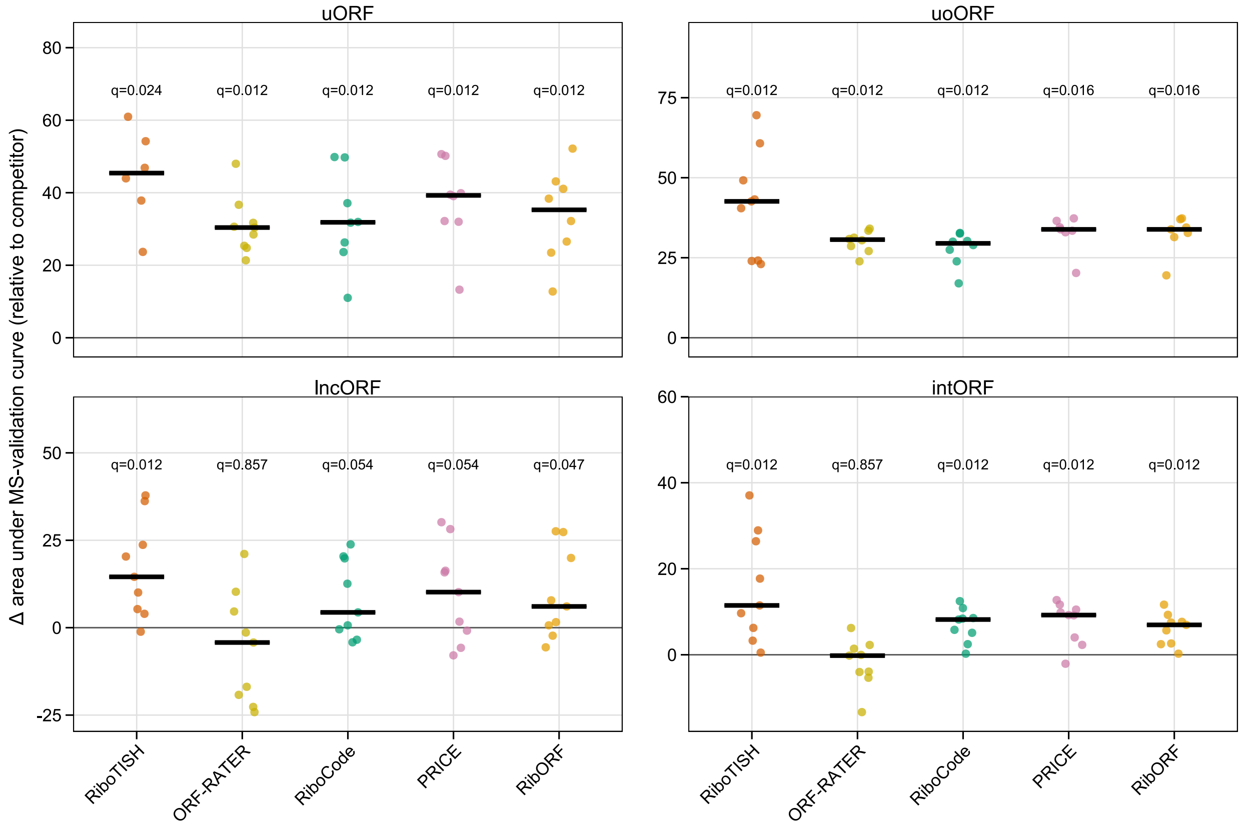

Supplementary Figure S16. Paired library-level comparison of the area under the MS-validation curve between RiboBA and competing tools. The summary statistic is the area under each tool's MS-validation curve—the cumulative fraction of MS-validated ncORFs against the number of top-ranked identifications—so that a larger area denotes higher MS-validation yield at matched ranking depth. Each point shows the per-library difference (RiboBA relative to competitor) for one of nine human Ribo-seq libraries, stratified by ncORF biotype (uORF, uoORF, lncORF, intORF); horizontal bars, median difference. q values are from one-sided paired Wilcoxon signed-rank tests (BH-corrected across all biotype–competitor pairs).

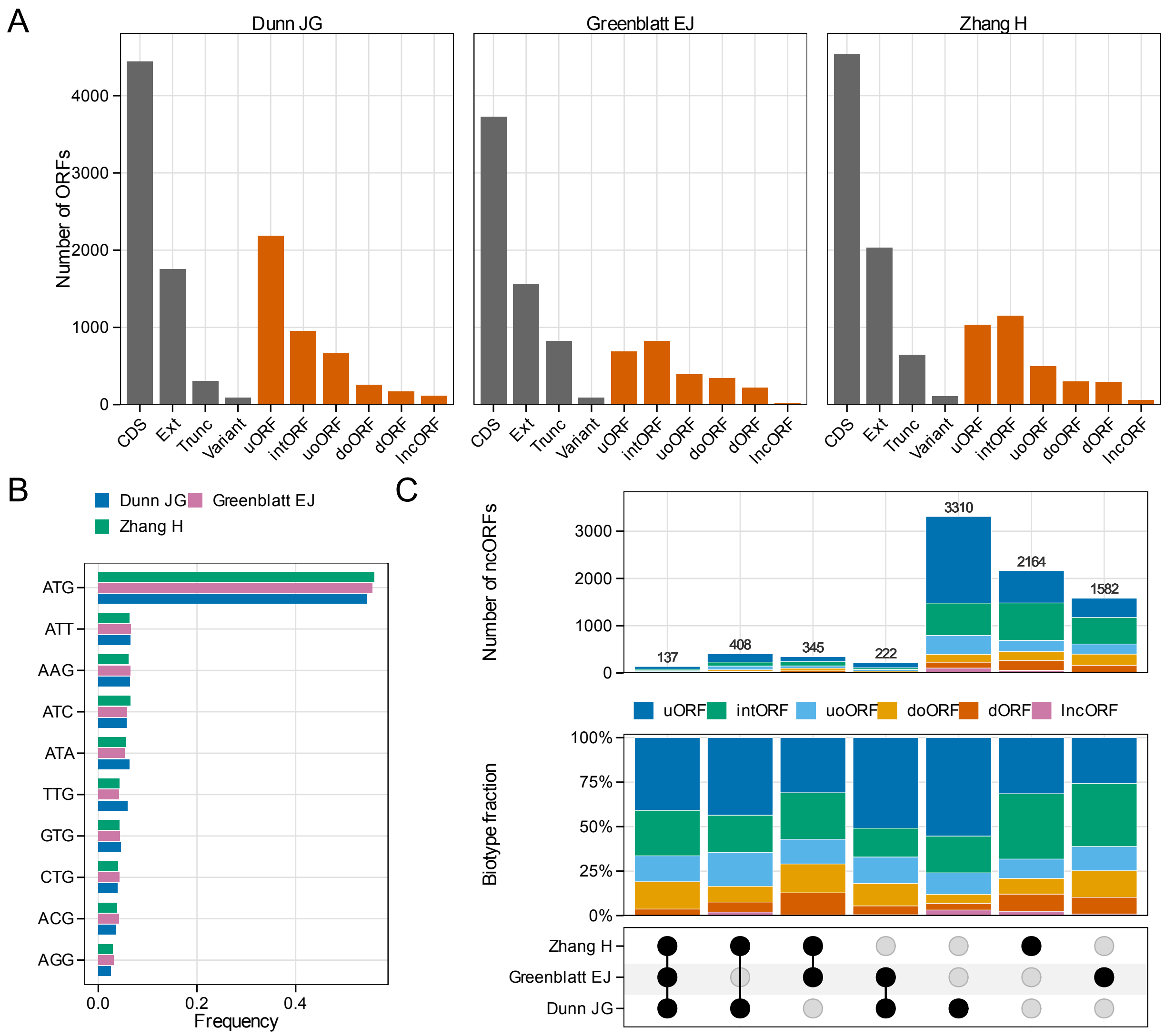

Supplementary Figure S17. Summary and reproducibility of identified ORFs in empirical *Drosophila* Ribo-seq datasets. Identified ORFs were compiled from three *Drosophila melanogaster* Ribo-seq datasets. **(A)** Total counts of identified ORFs by biotype in each dataset, with ncORF biotypes highlighted and lncORF denoting ncORFs originating from long non-coding RNA transcripts. **(B)** Start-codon usage across datasets, shown as the frequency of each initiator among identified ncORFs. **(C)** Reproducibility of RiboBA-identified ncORFs across the three datasets. ORFs sharing a stop-site position are counted as the same ORF, allowing alternative start sites.

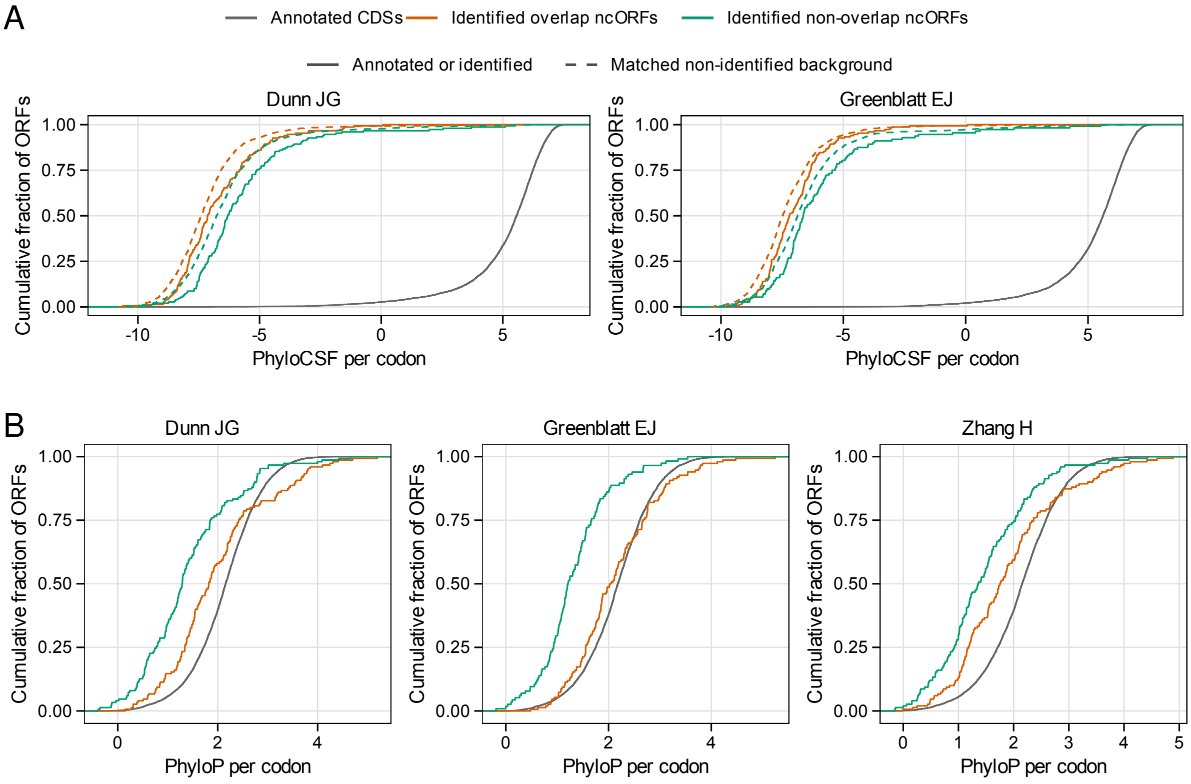

Supplementary Figure S18. PhyloCSF and phyloP distributions of RiboBA-identified *Drosophila* ncORFs. Empirical cumulative distribution functions (ECDFs) of per-codon PhyloCSF and phyloP scores are shown for annotated CDSs and top-ranked ncORFs (top 50 per biotype ranked by footprint support). ncORFs are grouped into overlapping (uoORF, intORF, or doORF) and non-overlapping (uORF, dORF, or lncORF) categories. **(A)** PhyloCSF ECDFs for the Dunn and Greenblatt datasets; dashed curves represent biotype-matched sequences from the same transcripts that were not identified as translated. Identified ncORFs were compared with biotype-matched background ORFs using one-sided Wilcoxon rank-sum tests (RiboBA-identified $>$ matched background; Dunn: $P = 1.97 \times 10^{-4}$ for overlapping and 3.01×10^{-6} for non-overlapping ncORFs; Greenblatt: $P = 8.14 \times 10^{-4}$ for overlapping and 4.43×10^{-3} for non-overlapping ncORFs). **(B)** phyloP ECDFs for the Dunn, Greenblatt, and Zhang datasets.

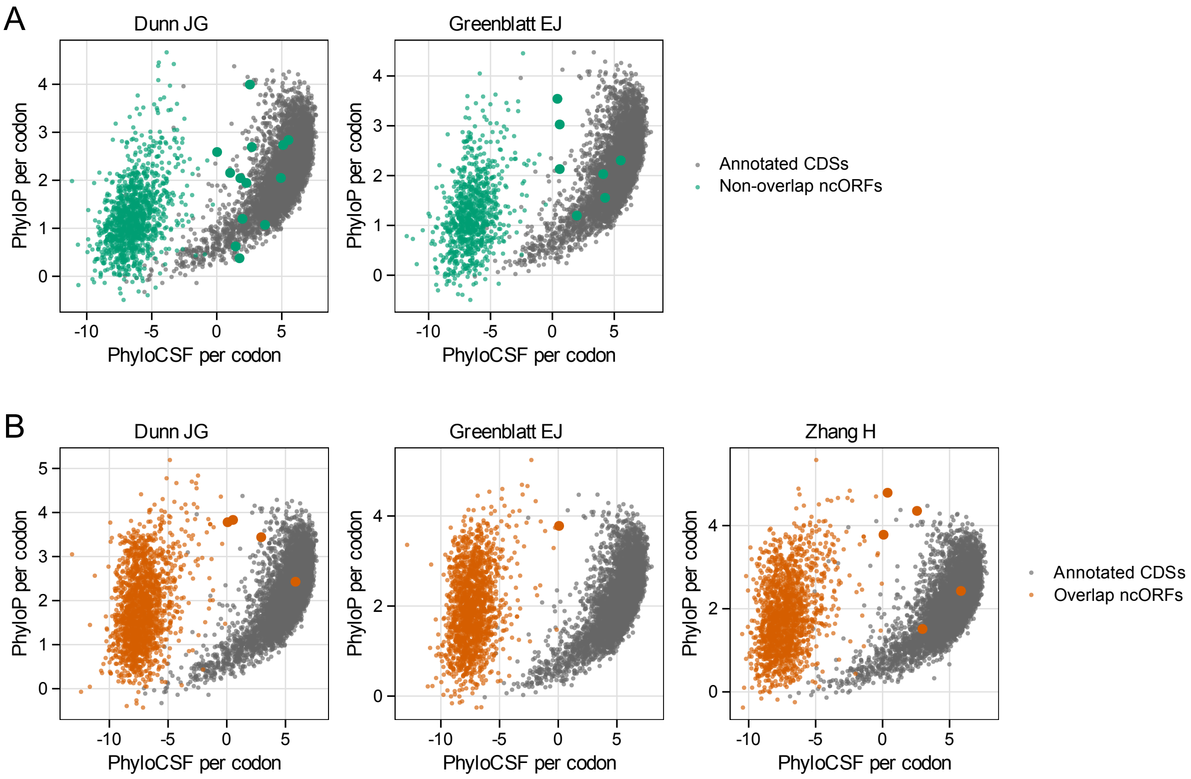

Supplementary Figure S19. Joint distribution of PhyloCSF and phyloP scores for RiboBA-identified *Drosophila* ncORFs. Scatter plots of per-codon PhyloCSF versus phyloP scores for annotated CDSs and identified ncORFs in empirical *Drosophila* Ribo-seq datasets. **(A–B)** ncORFs are shown separately for **(A)** non-overlapping (dORF, lncORF, and uORF) and **(B)** overlapping (doORF, intORF, and uoORF) categories. Points represent the top 1,000 identified ncORFs per biotype ranked by footprint support; ncORFs with positive PhyloCSF scores (PhyloCSF $>$ 0) are highlighted.

Supplementary Note

1. Ribosome-geometry constraints and P-site initialization

RiboBA employs a discrete ribosome footprint layout parameterized by (L_5, S_5, S_3, L_3), where L_5 and L_3 specify the admissible distance ranges between the P-site and the 5′ and 3′ termini, respectively, and S_5 and S_3 define protected offsets. We initialize these parameters based on the minimum retained footprint length and the observed length distribution. To optimize the layout, we optionally apply an iterative adjustment heuristic that shifts boundary values by one nucleotide when the inferred distance-dependent steric-hindrance profiles concentrate probability mass at the most distal admissible distances.

Given the footprint layout parameters (L_5, S_5, S_3, L_3), each footprint class $r \equiv (s, \ell)$ defines a feasible set $\mathcal{P}(r)$ of candidate P-site positions. A position p is included in $\mathcal{P}(r)$ if it satisfies the discrete end-to-P-site distance constraints for length ℓ, subject to two biological constraints: p must be in-frame with and contained within the annotated CDS. These feasible sets $\mathcal{P}(r)$ specify the candidate P-site positions for the mixture model in Eq. (1) and define the support over which the conditional distribution $\Pr(r \mid p; \Theta)$ in Eq. (2) is normalized.

Specifically, δ_ℓ is determined by aggregating the 5′ termini of reads of length ℓ into a metagene profile around annotated start codons. Within an admissible range (typically 12–15 nt), δ_ℓ is defined as the distance that maximizes the read count, effectively representing the most frequent offset for that fragment length. For each footprint class $r \equiv (s, \ell)$, we then assign an initial P-site position as:

$$p = s + \delta_\ell,$$

which provides the baseline ribosome occupancy profile for initializing the probabilistic model.

2. Estimating the 5′ non-templated addition distribution

We model 5′ non-templated additions using a library-specific distribution $\alpha = (\alpha_A, \alpha_C, \alpha_G, \alpha_T, \alpha_\varnothing)$, representing the probabilities of adding one of the four nucleotides or no addition ($\alpha_\varnothing$). Parameter estimation is based on three assumptions: (i) first-base mismatches at the 5′ terminus of addition-eligible reads arise predominantly from non-templated additions

rather than sequencing errors or alignment artifacts; (ii) the composition of added bases is independent of the reference nucleotide at the first aligned position; and (iii) additions are restricted to at most one extra nucleotide.

Let $x \in \{A, C, G, T\}$ denote the reference nucleotide at the first aligned position and $a \in \{A, C, G, T\}$ denote the first sequenced base of the read. For non-templated addition-eligible reads exhibiting a mismatch ($x \neq a$), we summarize the observed counts in a mismatch table $M_{x,a}$. We first estimate the conditional distribution of added nucleotides given an addition event, denoted by $\tilde{\alpha} = (\tilde{\alpha}_A, \tilde{\alpha}_C, \tilde{\alpha}_G, \tilde{\alpha}_T)$, where $\tilde{\alpha}_a \geq 0$ and $\sum_a \tilde{\alpha}_a = 1$. The expected probability $p_{x\to a}(\tilde{\alpha})$ of observing base a as a mismatch at reference position x, conditional on an addition event, is defined as:

$$p_{x\to a}(\tilde{\alpha}) = \frac{\tilde{\alpha}_a}{1 - \tilde{\alpha}_x}, \qquad a \in \{A, C, G, T\} \setminus \{x\}.$$

The corresponding observed mismatch fractions are calculated as:

$$\widehat{p}_{x\to a} = \frac{M_{x,a}}{\sum_{b\neq x} M_{x,b}}.$$

We then obtain the estimate $\widehat{\tilde{\alpha}}$ by minimizing the squared difference between the expected and observed fractions on the log scale:

$$\widehat{\tilde{\alpha}} = \arg\min_{\tilde{\alpha}} \sum_x \sum_{a\neq x} \left[\log \widehat{p}_{x\to a} - \log\left(\frac{\tilde{\alpha}_a}{1 - \tilde{\alpha}_x}\right)\right]^2,$$

subject to $\tilde{\alpha}_a \geq 0$ and $\sum_a \tilde{\alpha}_a = 1$.

In the second step, we recover the overall addition rate. Let π_x denote the background frequency of reference nucleotide x among the mapped 5′ positions of addition-eligible reads, and let m denote the observed fraction of such reads exhibiting a mismatch at the first aligned position. Under this model, the mismatch fraction satisfies:

$$m = \pi\Big(1 - \sum_x \pi_x \tilde{\alpha}_x\Big),$$

where π represents the probability of a non-templated addition event. This relationship yields the estimator:

$$\widehat{\pi} = \frac{m}{1 - \sum_x \pi_x \widehat{\tilde{\alpha}}_x}.$$

We then combine the estimated overall addition rate $\widehat{\pi}$ with the conditional composition $\widehat{\tilde{\alpha}}$

to obtain the five-category addition distribution $\widehat{\alpha}$:

$$\widehat{\alpha}_{\varnothing} = 1 - \widehat{\pi}, \qquad \widehat{\alpha}_a = \widehat{\pi}\,\widehat{\widetilde{\alpha}}_a, \quad a \in \{A, C, G, T\}.$$

The distribution $\widehat{\alpha}$ is used to correct 5′ terminus positions prior to EM-like fitting by redistributing reads across footprint classes. For reads with a first-base mismatch, the model deterministically attributes the mismatch to a non-templated addition, and their contribution is shifted to the corrected footprint class. For reads where the first base matches the reference, the presence of a non-templated addition is treated as a latent variable. Using the estimated model, we compute the posterior probability of a 5′ addition for each read and partition its contribution between the original and shifted footprint classes, ensuring that the total count $\sum_r \widetilde{y}_r = \sum_r y_r$ is preserved.

3. Inferring cleavage-related parameters θ

With λ fixed, we update θ by matching the observed footprint-class counts with model-predicted expectations. Let $\widehat{y}_r(\theta)$ denote the expected count for footprint class r under the generative model defined in Eq. (1), evaluated using current parameters; when decoupling ligation effects, the ligation term $L(r;\beta)$ is set to 1. To stabilize fitting across varying count magnitudes, we compare the observed counts y_r and transformed expectations $\widehat{y}^*_r(\theta)$, where:

$$\widehat{y}^*_r(\theta) = k_r \widehat{y}_r(\theta),$$

and k_r is an optional multiplicative factor that incorporates estimated terminal ligation efficiencies (e.g., $k_r = L(r;\widehat{\beta})$). We then minimize a robust discrepancy between these observed and expected counts:

$$\widehat{\theta} = \arg\min_{\theta \in \Omega_\theta} \sum_{r \in \mathcal{R}} \frac{\left|\widehat{y}^*_r(\theta) - y_r\right|}{\widehat{y}^*_r(\theta) + \left(\widehat{y}^*_r(\theta)\right)^2/10}, \tag{1}$$

where Ω_θ imposes box constraints on the nuclease-bias parameters ($10^{-6} \leq b_x \leq 1 - 10^{-6}$ and $10^{-8} \leq h_t(d) \leq 1 - 10^{-6}$ for all nucleotides x and distances d). The objective function in Eq. (1) is optimized using constrained derivative-free numerical routines under Ω_θ.

4. Estimation of ligation parameters

Following the stabilization of (λ, θ), we attribute the remaining systematic discrepancies between observed footprint counts and baseline expectations to sequence-dependent ligation efficiency. Let $\widehat{y}_r^{(0)}$ denote the baseline expected count for footprint class r, computed under current (λ, θ) with ligation effects held constant (i.e., $L(r;\beta) = 1$). We then employ a regression model with a log-link and a structural offset:

$$\log(y_r) = \log(\widehat{y}_r^{(0)}) + \eta_0 + \eta_5\big(k_5(r)\big) + \eta_3\big(k_3(r)\big) + \varepsilon_r,$$

where $k_5(r)$ and $k_3(r)$ represent the 5′ and 3′ terminal k-mers of footprint class r, η_0 is a global intercept, and $\eta_5(\cdot)$ and $\eta_3(\cdot)$ denote additive terminal k-mer effects on the log scale. The fitted values define a multiplicative correction factor:

$$L_{\text{reg}}(r) = \exp\Big\{\eta_0 + \eta_5\big(k_5(r)\big) + \eta_3\big(k_3(r)\big)\Big\}$$

This factor is mapped to the multiplicative terminal k-mer model described in the Main Text by setting:

$$\beta_5(u) \propto \exp\{\eta_5(u)\}, \qquad \beta_3(v) \propto \exp\{\eta_3(v)\},$$

where normalizing constants are chosen such that $0 < \beta_5(u) < 1$ and $0 < \beta_3(v) < 1$ for all terminal k-mers u and v. To ensure robust estimation for rare terminal contexts, the regression is performed on a balanced subset of footprint classes.

5. ORF identification using alternative tools

The specific parameters and pipelines used for each tool are summarized below; detailed scripts and configuration files for all benchmarking analyses are provided at `https://github.com/Bai-JunYu/ORF-calling`.

RibORF (version 2.0) was executed on TopHat2 (version 2.1.1) alignments generated using Bowtie2 (version 2.5.1) with a maximum of two mismatches. Candidate ORFs with a minimum length of 6 codons (including both ATG and non-ATG starts) were identified using `ORFannotate.pl`. P-site offsets were inferred via `readDist.pl` and refined with `offsetCorrect.pl`. Final translated ORFs were identified per sample using `ribORF.pl`.

RiboCode (version 1.0.0) utilized a reference transcriptome constructed from Ensembl GTF and GRCh38 FASTA files via `prepare_transcripts`. Reads were aligned using

STAR (version 2.7.10b) in end-to-end mode ($\leq$2 mismatches) with unique mapping enforced. P-site offsets and valid read lengths (19–42 nt; $f_0 \geq 0.4$) were inferred from metagene plots, and translated ORFs were subsequently identified.

PRICE, as implemented in GEDI (version 1.0.6), utilized STAR (version 2.7.10b) to generate coordinate-sorted BAM files, allowing up to 2 mismatches and 20 multimapping alignments. Following genome indexing, ORFs were identified using the `Price` module, and outputs were converted from CIT to BED format using `ViewCIT`.

ORF-RATER was applied to the coordinate-sorted BAM files used for PRICE. Transcript annotations were converted to BED12 format and redundant isoforms were pruned. Candidate ORFs were generated using `find_orfs`, followed by P-site inference via `psite_trimmed.py`. Translation was modeled using `regress_orfs.py` and quantified from the resulting BED outputs.

RiboTISH (version 0.2.7) was executed on the same BAM files as PRICE. Footprint periodicity and valid read lengths (19–42 nt) were assessed using a periodicity threshold of 0.4. ORFs were identified using `ribotish predict` with the `--framebest` option to prioritize the most periodic frame; the `--alt` flag was enabled to allow for non-ATG initiation.
